# A comprehensive mortise and tenon structure selection method based on Pugh’s controlled convergence and rough Z-number MABAC method

**DOI:** 10.1371/journal.pone.0283704

**Published:** 2023-05-18

**Authors:** Bin Shang, Zhe Chen, Qing Ma, Yuhang Tan

**Affiliations:** 1 School of Architecture and Design, China University of Mining and Technology, Xuzhou, 221116, China; 2 Shandong Jiaotong University, Jinan, 250357, China; 3 Advanced Manufacturing Technology Research Center, Shandong University of Science and Technology, Qingdao, 266590, China; Faculty of Applied Management, Economic and Finance, Belgrade, University Business Academy in Novi Sad, SERBIA

## Abstract

Mortise and tenon joints are widely used in the building and furniture industries because of their excellent mechanical and eco-friendly properties. In real-life cases, there are usually many available alternative structures for a joint area, it is a challenge to select a proper structure from massively available alternatives. This paper aims to select a proper multiple attribute decision-making method based on massive alternatives and unreliable, uncertain and subjective information. Pugh’s controlled convergence, rough number, Z-number, consistency theory and Shannon entropy are integrated and proposed an improved rough Z-number Multi-Attributive Border Approximation Area Comparison (MABAC) method. Firstly, Pugh’s controlled convergence is a selection method, simple and rapid, presented in the first phase to eliminate most of the alternatives. In the second phase, an integrated method is proposed. The consistency theory, distance measurement and the Z-number are initially aggregated to calculate the expert weight. The entropy method is then presented to determine the criteria weight. The alternatives are then ranked and the optimal mortise and tenon joint is selected based on the rough Z-number MABAC method. A real-life case is presented, and the proposed method is implemented in the joint of a bucket cabinet. Finally, the efficiency and effectiveness of the proposed method are proved by the case, sensitivity analysis and related comparisons.

## 1. Introduction

As a natural and renewable material, timber has been widely used in the building and furniture industries [1]. In these industries, many wooden parts are joined by connectors such as glue and screws. However, there are drawbacks to using these additives. Disassembly and recycling processes are not convenient, and some of the connectors are not eco-friendly [2]. Mortise and tenon joints are a traditional form of adhesion, and the parts are connected by the cyclic loading generated by themselves. Moderate deformations of the parts increase the strength of the structures, giving the structures excellent energy dissipation and bending resistance ability [3]. Semi-rigid joint structures cause wood to be more durable.

With timber a traditional material in China, there are plenty of applications for mortise and tenon structures. Mortise and tenon joints are formed by concave-convex structures, and the connecting parts and their structural modes are changeable. There are 10 main kinds of mortise and tenon structures [4], and each kind has many mortise and tenon joints. We have collected over 400 mortise and tenon structures and developed over 200 new structures. Each structure has its advantages and drawbacks. The differences across the structures are mainly due to six reasons: structural strength, size, fabricating time and cost, production equipment condition, aesthetics of appearance, and durability in the life cycle [5,6]. For example, the straight tenon joint and the dovetail tenon joint are two critical structures amongst over 80 mortise and tenon square T-joints. Compared to the dovetail tenon structure, the straight tenon structure has advantages in fabricating time and equipment requirements, but the tensile strength of the structure is weaker. Another example is the straight tenon structure made with an embedded tenon. Its production time and cost are less than for the traditional straight tenon, but because of the structural separation, it is difficult for the tenon part to expand and contract with the whole structure, so its mechanical strength and structural life are weaker than the traditional straight tenon structure. As a result, various mortise and tenon joints provide designers with more options.

In real-life cases, designers are expected to select the most suitable structure according to complicated requirements. However, this often does not occur. Even though the mortise and tenon joint selection process is very critical in the building and furniture industries, the producers inevitably use an empirical and stylized approach due to differences in cognition and personal ability, meaning decisions rely on a designer’s individual experience.

The motivation of this paper is to establish a mortise and tenon joint selection model dealing with the various complex joint types, making the selection process more scientific and reasonable, considering the requirements in multiple fields. Problems like this of selecting an optimal alternative from several options under complicated rules are defined as Multiple Attribute Decision Making (MADM) issues. Effective and efficient are two critical requirements for MADM problems. To meet the requirements, Pugh’s controlled convergence method and the rough Z-number Multi-Attributive Border Approximation Area Comparison method (MABAC) are combined in our study. Firstly, the basic information is obtained from the experts’ judgements, they are subjective, unreliable and uncertain. The rough number has shown its strength in dealing with subjectivity while Z-number is good at handling unreliability and uncertainty. The integration of rough numbers and the Z-number MABAC method can ensure the effectiveness of the decision-making process. Although it is an excellent method for MADM problems with subjective, unreliable and uncertain information, the quality of the decision-making process cannot be guaranteed due to the heavy workload of the DMs for dozens of alternatives. Pugh’s controlled convergence method is a simple method for alternative rapid elimination and can exclude the inapposite alternatives which not meet the requirements. Hence, in this paper, an integrated mortise and tenon structure selection method is proposed with two phases. First, Pugh’s controlled convergence method is implemented to eliminate unsuitable alternatives rapidly. In this phase, most structures which are inappropriate for the project are eliminated. Then, a selection method based on the rough Z-number is proposed. In this step, the remaining mortise and tenon joints are ranked by a MABAC method, and a proper structure for the case is selected.

This article makes three contributions:

1. It improves the rough Z-number MABAC method. The Pugh’s decision matrix, rough Z-number, consistency theory, Shannon entropy and MABAC method are integrated for product structure selection. The expert weight determination, criteria weight calculation and alternative ranking identification process of mortise and tenon joint selection are conveniently combined.
2. It provides an integrated Z-number and rough number for the uncertain, unreliable and subjective decisions made by experts. The linguistic information given by the experts has the characteristics of being uncertain, unreliable and subjective [7]. On one hand, given the rough number’s excellent performance in manipulating subjectivity, it is widely used in alternative selection in product design [8]. On the other hand, the Z-number is used to solve problems considering uncertainty and reliability [9,10]. Hence, the rough Z-number is used in this study.
3. It introduces a comprehensive mortise and tenon joint selection model based on Pugh’s controlled convergence method and rough Z-number MABAC method. The integrated model is used for decision-making with a large number of alternatives, both the effective and efficient are considered in this process. A real-life case study and comparisons are conducted to identify the validity of the proposed methodology.

The remainder of the paper is structured as follows. Section 2 reviews relevant decision-making theories and approaches, including the MADM, Pugh’s controlled convergence method, rough Z-number and the MABAC method. Section 3 provides some definitions from the relevant theories. Section 4 proposes mortise and tenon structure selection methods based on many alternatives. In section 5, a real-life example and some comparisons are applied to validate the proposed method. Section 6 concludes with sensitivity analysis, comparison and some important findings.

## 2. Literature review

This section has four parts. Section 2.1 reviews the related theories of MADM. Section 2.2 reviews Pugh’s controlled convergence, which is used to eliminate the inappropriate structure in Phase 1. Section 2.3 and Section 2.4 are about the rough Z-number and the MABAC method which significantly improve the precision of Phase 2.

### 2.1 Multiple attribute decision making

In the past decades, concept selection methods such as Quality Function Deployment (QFD) [11], SWOT analysis [12], Pugh’s controlled convergence [13] and screening matrix [14] are easy to operate, and are applicable for conditions requiring simple and fast convergence. Once the alternatives and the attributes become complex, some more complicated mathematical approaches emerge. MADM, as a successful decision-making method, has shown excellent performances in various complex design concept selection fields. In MADM problems, the Decision Maker (DM) or DMs are asked to evaluate a finite number of pre-defined alternatives according to a set of conflicting assessment attributes [15]. In other words, the MADM method in design concept selection is the result of a compromise of various attributes, with the alternative selected as the one that best balances the relationships among the attributes [16]. As the situations vary, various MADM methods are introduced in design concept selection. Analytic Hierarchy Process (AHP) is one of the most popular MADM methods in design concept selection [17]. Pairwise comparisons between alternatives are made by experts, and the ranking of the alternative is determined by its performance in pairwise comparisons and the corresponding weight in hierarchical structure [18]. VlseKriterijumska Optimizacija I KOmpromisno Resenje (VIKOR) and Technique for Order Performance by Similarity to Ideal Solution (TOPSIS) are compromise-based methods. The main idea of the methods based on compromise theory is comparing the distance between each scheme and the pre-determined ideal solution [19,20]. Recently, another compromise-based method, Measurement Alternatives and Ranking based on COmpromise Solution(MARCOS) is proposed and shown its advantages in several fields including material selection for aircraft design [21,22]. The ELimination Et Choice Translating REality (ELECTRE) and the preference ranking organizational method are based on hierarchical relationships. By evaluating decision attributes, DMs establish a decision-making framework of the hierarchical relation of alternative solutions to realize the purpose of ranking the solutions [23,24]. Some other methods introduce proper aggregation operators and rank the alternatives by similarity or distance [25,26].

In the wood product field, applications of MADM are mainly focused on wood material selection. Lipušček [27] proposed a MADM model based on the hierarchical structure to rank the environmental impact of the wood materials. Sibagariang [28] proposed a Bayes method to determine the best wood quality for cabinet production. Peng [29] integrated the generalized Data Envelopment Analysis (DEA) method and the TOPSIS method for furniture wood selection. Cui [30] also utilized a hybrid method for outdoor wooden furnishing selection. In his study, a multi-factor fuzzy comprehensive evaluation method is proposed based on trapezoidal fuzzy AHP. Although the applications are available for the specific case, the uncertainty, reliability and subjectivity of the information obtained from the experts is not completely considered.

To mitigate the effect of the uncertain environment in MADM problems, fuzzy set integrated methods are widely applied in design concept selection. Zhai [31] first used rough numbers to illustrate the vagueness of the DMs’ judgements and introduced a design concept selection method based on the rough numbers and QFD. Since then, Rough-VIKOR [32], Rough-TOPSIS [8], Rough-AHP [17], and other rough number-based methods have become very popular in this field [33]. As well as the rough number, other fuzzy logic algorithms such as fuzzy set [34], soft set [35] and Z-number [36] are used in product concept selection.

### 2.2 Pugh’s controlled convergence

Pugh’s controlled convergence is one of the decision-making methods introduced by Pugh [37]. The Pugh decision matrix is established to evaluate the ranking of the alternatives. The columns and the rows of Pugh’s matrix represent the design alternatives and the decision attributes. Symbols +, − or *S* are entered in the matrix cells, corresponding to better, worse or same at satisfying the decision attribute compared with other alternatives. The sum of the matrix by column shows the priority of the alternative. The design schemes can be compared by the column sums. As an alternative fast selection method, Pugh’s controlled convergence is simple and easy to do. Mia [38] investigated different sustainable techniques applied in the machining of hardened steel, and Pugh’s decision matrix was built to rank the relative techniques. Kim [39] also applied Pugh’s matrix to determine the impact location for composite plate in different conditions. Li [40] integrated a modified Pugh’s decision matrix, QFD and the KANO model to evaluate and rank refrigerator design concepts by the requirements of elderly users, and Pugh’s matrix in that study is similar to the weighted sum method matrix. Zhu [41] integrated the AHP, QFD and Pugh’s matrix in a similar way, and Pugh’s matrix is implemented to eliminate the alternatives which are obviously not suitable for the product R&D. The studies mentioned above show the efficiency of Pugh’s controlled convergence. The alternatives can be converged quickly using Pugh’s matrix, which is beneficial in a rapid development environment. However, as a semi-qualitative MADM method, Pugh’s controlled convergence lacks the vector weight of the alternatives [42]. Hence, the method is not as accurate as complicated methods.

### 2.3 Rough Z-number

Although the DMs are selected from experts who are very familiar with the alternatives, the complex structures and criteria make the experts’ decisions complicated [43]. Hence, Z-number is proposed to better measure the reliability of linguistic information. It is formed as *Z* = (*A*,*B*), where *A* denotes the preference of the expert, while *B* represents the possibility of *A*. To better understand Z-number, research has been implemented in several aspects. Extended Z-numbers including Z*-number [44], Z-advanced number [45] and Z^+^-number [46] are implemented in MADM problems. Meanwhile, as Z-numbers are not easy to solve [47], scholars started to convert Z-numbers into Triangular Fuzzy Numbers(TFNs) and trapezoidal fuzzy numbers, solving the problems by fuzzy numbers [48]. Kang [49] investigated the operation of Z-numbers, and proposed a transforming rule, where Z-numbers are transformed into fuzzy numbers and trapezoidal fuzzy numbers. Božanić [50] converted the Z-number into TFNs, combined the level-based weight assessment method and multi-attributive ideal-real comparative analysis method in camp location selection. Garg [51] also proposed a method to convert Z-numbers into granulated Z-numbers. Other scholars integrated the Z-number and MADM methods to improve the reliability of their studies. Peng [9] improved the MULTIMOORA method, and proposed a Z-number Choquet integral projection operator, developed a MADM method with pairwise evaluation in a Z-number environment. Cheng [52] proposed a distance-based method that integrated TOPSIS and Z-number. Jia [53] introduced a hybrid MADM method based on Z-numbers, interval-valued intuitionistic fuzzy sets and trapezium clouds. In these studies, Z-number shows its powerful ability to deal with uncertainty and reliability conditions [54]. However, it cannot convey the subjectivity of the DMs in mortise and tenon structure selection. To accommodate subjectivity, uncertainty and reliability, the rough number is combined with Z-number in this study.

### 2.4 Multi-Attributive border approximation area comparison method

Since the MABAC method was introduced by Pamučar, it has attracted widespread attention from various fields [33]. Since then, MABAC started to solve Z-number based MADM problems. Huang [33] introduced a MADM method based on Z-cloud rough numbers and the best-worst method and MABAC. A real-life case of refrigerator design is presented and some comparisons are analyzed to elaborate and validate the method. Shen [55] introduced a directed distance and regret theory and proposed an extended Z-number based MABAC method in problem-solving, and used a case about regional circular economy development program selection to show the efficiency and feasibility of the proposed method. Zhu [56] proposed a rough Z-number Analytic Hierarchy Process (AHP) and a rough Z-number MABAC method where the rough Z-number AHP is presented to determine the criteria weight, then the alternatives are ranked according to a rough Z-number MABAC method.

**Fig 1:**
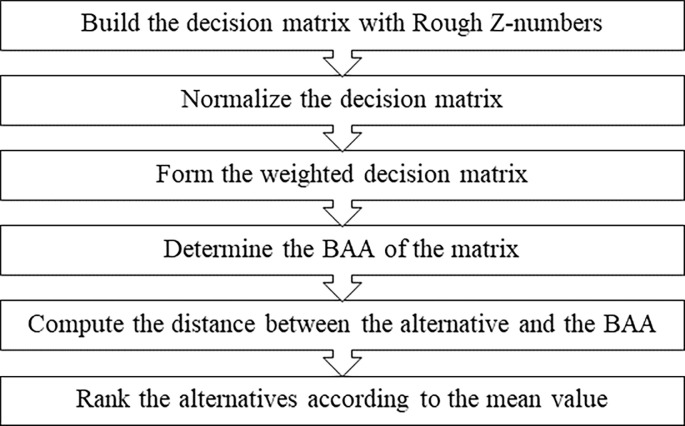
The MABAC method based on rough Z-numbers.

The process of the rough Z-number MABAC is shown in Fig 1. First, the rough Z-number decision matrix is determined based on the linguistic information provided by the DMs. Second, the rough Z-number decision matrix is normalized by a max-min method. Third, by integrating with the criteria weight matrix in a proper way, a weighted decision matrix is constructed. Fourth, the values of the attribute functions are computed for the alternatives. Fifth, the distance between the attribute and the Border Approximation Area (BAA) is calculated. Sixth, the alternatives are then ranked by the distances.

**Fig 2:**
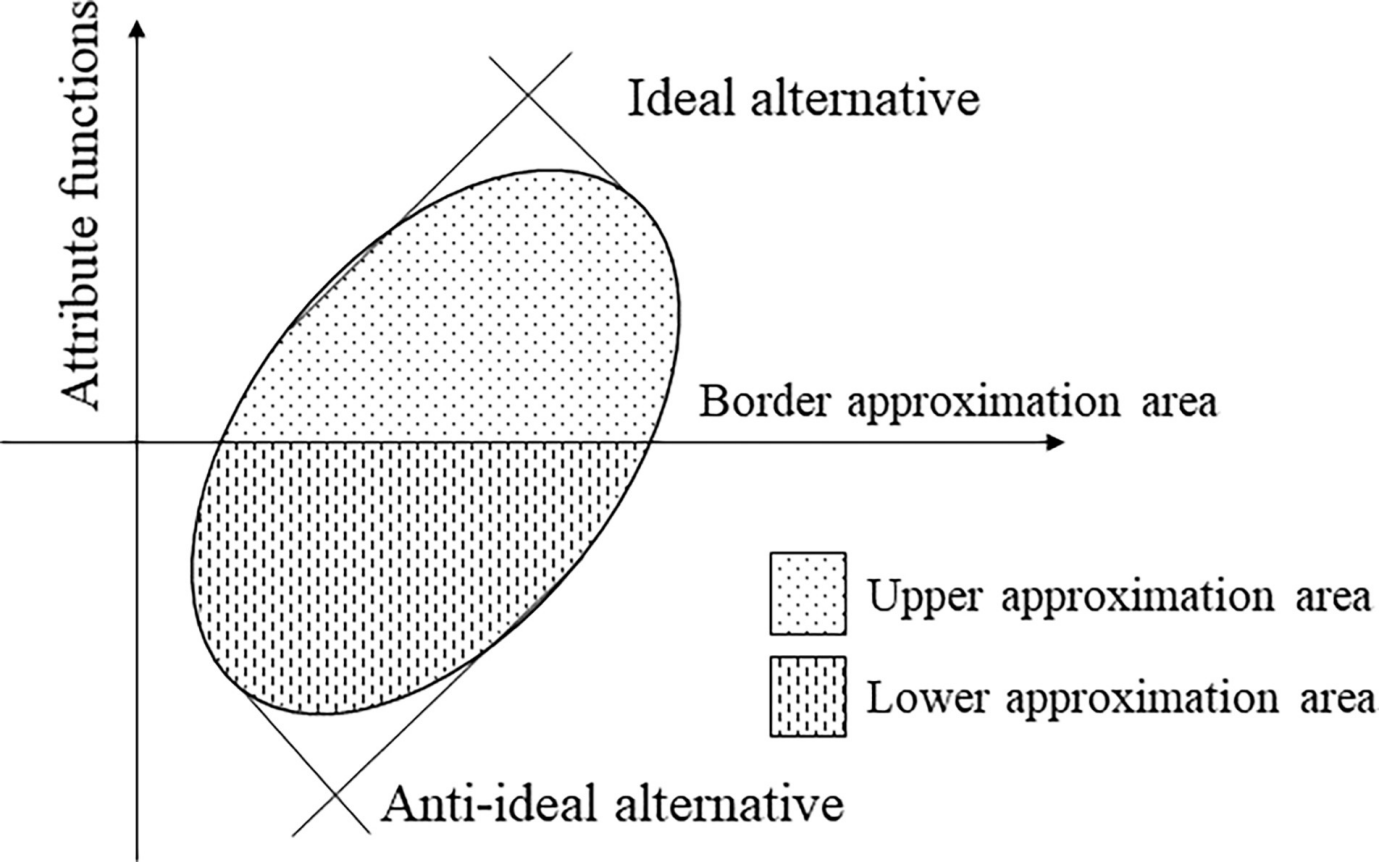
The upper and the lower approximation area.

The schematic diagram is shown in Fig 2. For each attribute, the BAA is calculated by the geometric mean of the related elements in the weighted decision matrix. Hence, if the alternative is in the upper approximation area, the attribute function is near or equal to the ideal option by this attribute. If the alternative is in the lower approximation area, it is near or equal to the anti-ideal alternative. Hence, the arithmetic mean can be used to aggregate the attribute functions. The rankings of the alternatives can be compared by arithmetic means.

## 3. Preliminaries

**Definition 1** [49] Assume $Z=(\tilde{A},\tilde{R})$ is a Z-number. $\tilde{A}$ and $\tilde{B}$ denote the restriction and reliability, respectively. Let $\tilde{A}=\{\left\langle x,u_{\tilde{A}}(x)\rangle |x\in [0,1\right]\},\tilde{B}=\{\left\langle x,\mu_{\tilde{R}}(x)\rangle |x\in [0,1\right]\}$, if $u_{\tilde{A}}(x)$ and $\mu_{\tilde{R}}(x)$ are both triangular membership functions. The Z-number can be converted by

$$\alpha =\frac{\int x\mu_{\tilde{R}}(x)dx}{\int \mu_{\tilde{R}}(x)dx}$$ (1)

$$\tilde{Z}=\left\{\langle x,\mu_{\tilde{Z}}(x)\rangle |\mu_{\tilde{Z}}(x)=\mu_{\tilde{A}}(\frac{x}{\sqrt{\alpha }}),x\in [0,1]\right\}$$ (2)

Where ∫ denotes an algebraic integration. Thus, the Z-number can be transferred into a triangular fuzzy number (TFN).

**Definition 2** [57] Assume $\tilde{R}=\{{\tilde{Z}}_{1},{\tilde{Z}}_{2},\ldots ,{\tilde{Z}}_{n}\}$ is a set of TFNs converted from Z-numbers. ${\tilde{Z}}_{i}=(a_{i},b_{i},c_{i})$ and ${\tilde{Z}}_{i}=(a_{j},b_{j},c_{j})$ are two elements in $\tilde{R},1\leq i\leq n$. The distance between ${\tilde{Z}}_{i}$ and ${\tilde{Z}}_{j}$ is

$$D({\tilde{Z}}_{i},{\tilde{Z}}_{j})=(d(a_{ij}),d(b_{ij}),d(c_{ij}))$$ (3)

Where d(*a*_*ij*_), d(*b*_*ij*_), d(*c*_*ij*_) can be determined by the equations below.


$$d(a_{ij})=\left\{ \begin{array}{c} max((a_{i}-c_{j}),0),\;b_{i}>b_{j}\\ max((a_{i}-c_{j}),(a_{j}-c_{i}),0),\;b_{i}=b_{j}\\ max((a_{j}-c_{i}),0),\;b_{i}<b_{j} \end{array}\right.$$ (4)

$$d(b_{ij})=|b_{i}-b_{j}|$$ (5)

$$d(c_{ij})=max(\left(c_{j}-a_{i}),(c_{i}-a_{j}\right))$$ (6)

The total distance between ${\tilde{Z}}_{i}$ and the other TFNs in $\tilde{R}$ is denoted by

$$D^{*}=\sum_{j=1,j\neq i}^{n}D({\tilde{Z}}_{i},{\tilde{Z}}_{j})=(\sum_{j=1}^{n}d(a_{ij}),\sum_{j=1}^{n}d(b_{ij}),\sum_{j=1}^{n}d(c_{ij}))$$ (7)

**Definition 3** [31] Let *U* be the universe, *R* be a set with *n* classes, denoted as *R* = {*C*_1_,*C*_2_,…,*C*_*n*_}, sorted in the manner of *C*_1_<*C*_2_<*C*_3_<⋯<*C*_*n*_. Assume *Y* is an arbitrary element of the set. ∀*Y*∈*U*, the lower approximation *Apr*(*C*_*i*_) and the upper approximations $\bar{Apr}(C_{i})$ satisfy

$$\left\{ \begin{array}{l} \underline{Apr}(C_{i})=\cup \{Y\in U/R(Y)\leq C_{i}\}\\ \bar{Apr}(C_{i})=\cup \{Y\in U/R(Y)\geq C_{i}\} \end{array}\right.$$ (8)

Where *C*_*i*_∈*R*. Hence, the lower limit *Lim*(*C*_*i*_) and the upper limit $\bar{Lim}(C_{i})$ in each class can be determined by

$$\underline{Lim}(C_{i})=\frac{1}{M_{L}}\sum R(Y)|Y\in \underline{Apr}(C_{i})$$ (9)

$$\bar{Lim}(C_{i})=\frac{1}{M_{U}}\sum R(Y)|Y\in \bar{Apr}(C_{i})$$ (10)

Where *M*_*L*_ and *M*_*U*_ are the number of elements in *Apr*(*C*_*i*_) and $\bar{Apr}(C_{i})$, respectively. The rough interval can be written as [*Lim*(*C*_*i*_), $\bar{Lim}(C_{i})$].

**Definition 4** [31] The arithmetic operations of rough numbers are

$${RN}_{1}\times {RN}_{2}=[A_{1},B_{1}]\times [A_{2},B_{2}]=[A_{1}\times A_{2},B_{1}\times B_{2}]$$ (11)

$${RN}_{1}+{RN}_{2}=[A_{1},B_{1}]+[A_{2},B_{2}]=[A_{1}+A_{2},B_{1}+B_{2}]$$ (12)

$${RN}_{1}\times k=[A_{1},B_{1}]\times k=[kA_{1},{kB}_{1}]$$ (13)

Where *RN*_1_ = [*A*_1_, *B*_1_] and *RN*_2_ = [*A*_2_, *B*_2_] are two rough numbers, *k* is a constant.

**Definition 5** [56] Let ${\tilde{Z}}_{i}=(a_{i},b_{i},c_{i})$ be a TFN converted from Z-number $Z_{i}=({\tilde{A}}_{i},{\tilde{R}}_{i})$, where 1≤*i*≤*n*. Assume *Z* be a set with *n* classes, denoted as

$$Z=\{Z_{1},Z_{2},\ldots ,Z_{n}\}=\{\left({\tilde{A}}_{1},{\tilde{R}}_{1}),({\tilde{A}}_{2},{\tilde{R}}_{2}),\ldots ,({\tilde{A}}_{3},{\tilde{R}}_{3}\right)\}$$ (14)

The converted TFNs can be defined as

$$\tilde{Z}=\{{\tilde{Z}}_{1},{\tilde{Z}}_{2},\ldots ,{\tilde{Z}}_{n}\}=\{\left(a_{1},b_{1},c_{1}),(a_{2},b_{2},c_{2}),\ldots ,(a_{n},b_{n},c_{n}\right)\}$$ (15)

According to Definition 1, rough numbers can be aggregated by the elements in $\tilde{R}$, denoted as $\left[\underline{Lim}(a_{i}),\bar{Lim}(a_{i})\right]$, $\left[\underline{Lim}(b_{i}),\bar{Lim}(b_{i})\right]$ and $[\underline{Lim}(c_{i}),\bar{Lim}(c_{i})],$ 1≤*i*≤*n*. The rough Z-number can be denoted as

$$RZ(\tilde{Z})=(\left[a_{i}^{-},a_{i}^{+}],[b_{i}^{-},b_{i}^{+}],[c_{i}^{-},c_{i}^{+}\right])$$ (16)

Where the *x*^−^ and *x*^+^ denote the lower and the upper limit, respectively.

**Definition 6** [56] Let ${RZ}_{1}=(\left[a_{1}^{-},a_{1}^{+}],[b_{1}^{-},b_{1}^{+}],[c_{1}^{-},c_{1}^{+}\right])$ and ${RZ}_{2}=(\left[a_{2}^{-},a_{2}^{+}],[b_{2}^{-},b_{2}^{+}],[c_{2}^{-},c_{2}^{+}\right])$ as two rough Z-numbers. The arithmetic operations of rough Z-numbers are

$${RZ}_{1}+{RZ}_{2}=(\left[a_{1}^{-}+a_{2}^{-},a_{1}^{+}+a_{2}^{+}],[b_{1}^{-}+b_{2}^{-},b_{1}^{+}+b_{2}^{+}],[c_{1}^{-}+c_{2}^{-},c_{1}^{+}+c_{2}^{+}\right])$$ (17)

$${RZ}_{1}\times {RZ}_{2}=(\left[a_{1}^{-}\times a_{2}^{-},a_{1}^{+}\times a_{2}^{+}],[b_{1}^{-}\times b_{2}^{-},b_{1}^{+}\times b_{2}^{+}],[c_{1}^{-}\times c_{2}^{-},c_{1}^{+}\times c_{2}^{+}\right])$$ (18)

$${RZ}_{1}\times k=(\left[a_{1}^{-}\times k,a_{1}^{+}\times k],[b_{1}^{-}\times k,b_{1}^{+}\times k],[c_{1}^{-}\times k,c_{1}^{+}\times k\right])$$ (19)

## 4. Proposed method

**Fig 3:**
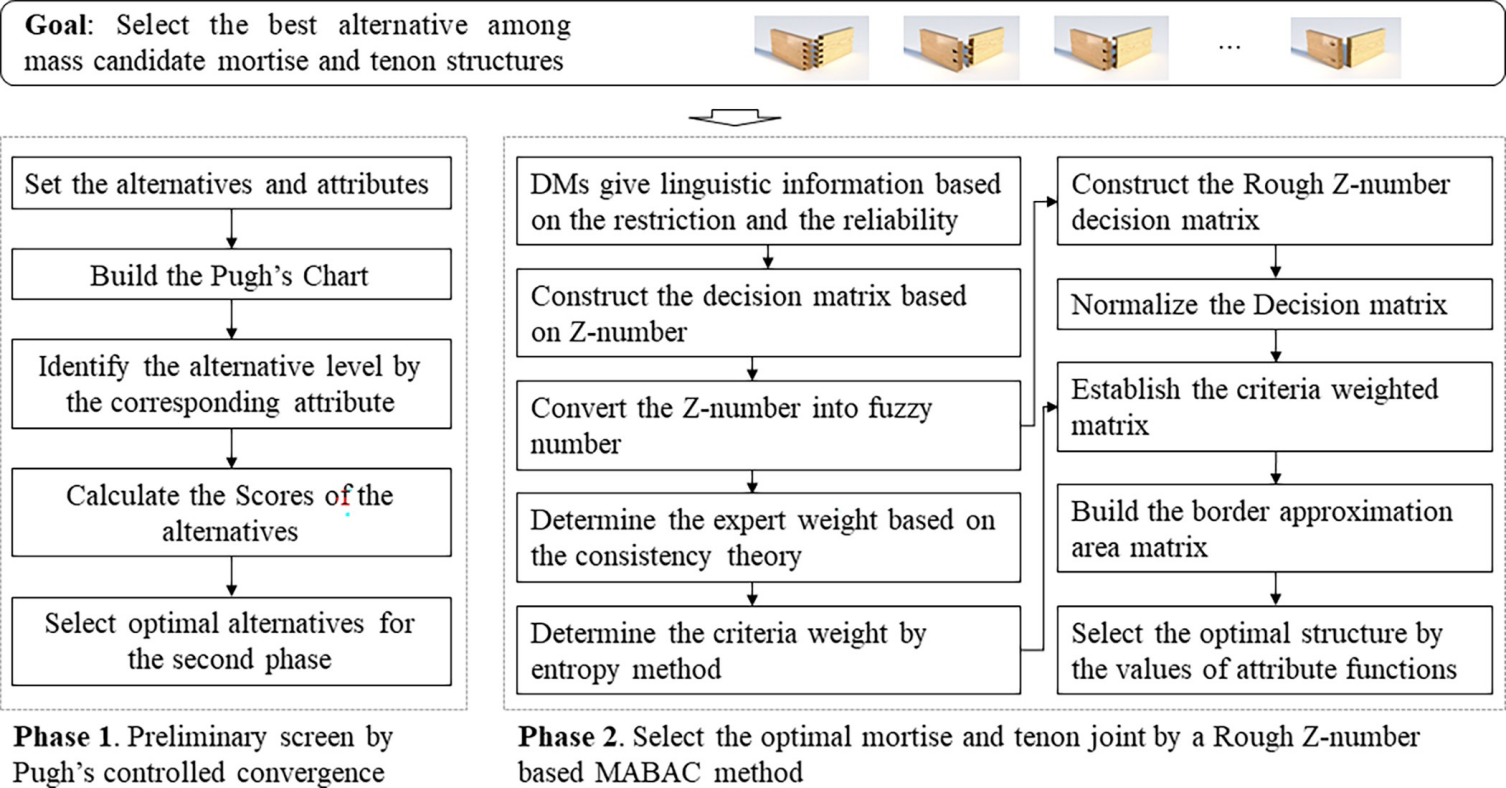
Schematic of the proposed mortise and tenon joint structure selection method.

To select an appropriate structure among massive mortise and tenon joints, a comprehensive method is proposed in our study. As is shown in Fig 3, the proposed method includes two phases. Pugh’s controlled convergence is applied in the first phase to eliminate the mortise and tenon joint structures which are obviously unsuitable for the specific case. Pugh’s controlled convergence is fast and does not need additional information, and thus is suitable for the preliminary screen. Then a much more precise method, the MABAC method based on the rough Z-number, is proposed to determine the optimal structure.

### 4.1 Preliminary structure screen by Pugh’s controlled convergence

In this phase, Pugh’s controlled convergence is adapted to eliminate the mortise and tenon joints which are obviously not available for the specific application scenario. The steps of this phase are shown as follows:

**Step 1**: Prepare the Pugh matrix. Assume there are *p* mortise and tenon joint structures as the alternatives, *q* attributes are selected as the evaluation criteria.

**Fig 4:**
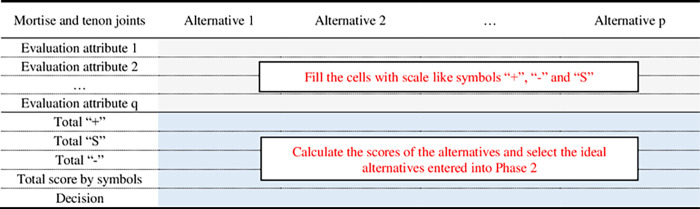
The Pugh decision matrix for mortise and tenon joints.

**Step 2**: Build Pugh’s matrix. Pugh’s matrix was then constructed as Fig 4 shows. The cells are filled with scale-like symbols “+”, “-” and “S”. The symbols are used to rate each design criteria of all the concepts against the specific utility. The symbol “+” represents the alternative that is superior according to the specific attribute. Correspondingly, the “-” and “S” represent the alternative is inferior and moderate to the specific attribute, respectively.

**Step 3**: The symbols “+”, “-” and “S” are scored as “1”, “-1” and “0”, respectively. The total score of each alternative is then ranked in descending order. The options with higher scores are then included in the second phase.

### 4.2 Select the optimal mortise and tenon joint by a rough Z-number based MABAC method

In this phase, a distance-based method is implemented to select an optimal structure from the mortise and tenon joint options. After the preliminary screening, assume there are *m* experts *E* = {*e*_1_, *e*_2_,…,*e*_*m*_}, *l* joints are selected as the alternatives, shown as *A* = {*A*_1_,…,*A*_*l*_}, *n* attributes are shown as *C* = {*c*_1_, *c*_2_,…,*c*_*n*_}. The details of the proposed method are as follows:

**Step 1:** Construct the decision matrix.

The experts are assigned to judge the alternatives by the criteria. In our study, experts’ judgements are made through linguistic information. During this process, each expert makes their decision based on their cognition. As the individual cognition mainly relies on their background including education and experience [58], the reliabilities of the experts’ judgements vary. The experts are required to make the risk assessment using linguistic variables in two parts, restriction information represents to the preference and the reliability represents to his confident level. Let Z-number $Z_{ij}^{\left(t\right)}=({\tilde{A}}_{ij},{\tilde{R}}_{ij})$ be the decision made by expert *e*_*t*_, where ${\tilde{A}}_{ij}$ and ${\tilde{R}}_{ij}$ represent the restriction and reliability of the expert’s decision.

The decision matrix of expert *e*_*t*_(*t* = 1,2,…,*m*) can be denoted by the equation below:

$$A^{\left(t\right)}=\left[\begin{array}{cccc} Z_{11}^{\left(t\right)} & Z_{12}^{\left(t\right)} & \cdots & Z_{1n}^{\left(t\right)}\\ Z_{21}^{\left(t\right)} & Z_{22}^{\left(t\right)} & \cdots & Z_{2n}^{\left(t\right)}\\ \vdots & \vdots & \ddots & \vdots \\ Z_{l1}^{\left(t\right)} & Z_{l2}^{\left(t\right)} & \cdots & Z_{ln}^{\left(t\right)} \end{array}\right],t=1,2,\ldots ,m$$ (20)

Accordingly, the decision matrices of other DMs can be denoted as *A*^(1)^, *A*^(2)^,…,*A*^(*m*)^.

**Step 2:** Convert the Z-number into a fuzzy number.

In this step, the Z-numbers are converted into TFNs according to Definition 1, where $Z_{ij}^{\left(t\right)}=({\tilde{A}}_{ij},{\tilde{R}}_{ij})$. The converted decision matrix of expert et *e*_*t*_ is

$$B^{\left(t\right)}=\left[\begin{array}{cccc} \left(a_{11}^{\left(t\right)},b_{11}^{\left(t\right)},c_{11}^{\left(t\right)}\right) & \left(a_{12}^{\left(t\right)},b_{12}^{\left(t\right)},c_{12}^{\left(t\right)}\right) & \cdots & \left(a_{1n}^{\left(t\right)},b_{1n}^{\left(t\right)},c_{1n}^{\left(t\right)}\right)\\ \left(a_{21}^{\left(t\right)},b_{21}^{\left(t\right)},c_{21}^{\left(t\right)}\right) & \left(a_{22}^{\left(t\right)},b_{22}^{\left(t\right)},c_{22}^{\left(t\right)}\right) & \cdots & \left(a_{2n}^{\left(t\right)},b_{2n}^{\left(t\right)},c_{2n}^{\left(t\right)}\right)\\ \vdots & \vdots & \ddots & \vdots \\ \left(a_{l1}^{\left(t\right)},b_{l1}^{\left(t\right)},c_{l1}^{\left(t\right)}\right) & \left(a_{l2}^{\left(t\right)},b_{l2}^{\left(t\right)},c_{l2}^{\left(t\right)}\right) & \cdots & \left(a_{ln}^{\left(t\right)},b_{ln}^{\left(t\right)},c_{ln}^{\left(t\right)}\right) \end{array}\right],t=1,2,\ldots ,m$$ (21)

**Step 3:** Determine the expert weight based on the consistency theory.

The expert weights illustrate the diversity among the DMs. Although the experts in product structure selection are arranged by the decision-making organizer, they may not be familiar with each other. Hence, they find it very difficult to provide accurate judgements to each other, and subjective weight determination is not available here.

The experts’ decisions are characterized as consensus and complex. Consistent conclusions must be made. The distance between an expert and the others reflects the consistency degree of the expert’s decision. Based on this theory, the consistency of expert *e*_*t*_ is defined as the reciprocal of the total distance between himself and the others.

According to Eq (3) to Eq (7), the total distance between expert *e*_*t*_ and the other experts can be calculated by

$$dis(t)=\sum_{k=1,k\neq t}^{m}\sum_{j=1}^{n}\sum_{i=1}^{l}D({\tilde{Z}}_{ijt},{\tilde{Z}}_{ijk})=(\sum_{k=1,k\neq t}^{m}\sum_{j=1}^{n}\sum_{i=1}^{l}d(a_{ij}^{\left(t\right)}),\sum_{k=1,k\neq t}^{m}\sum_{j=1}^{n}\sum_{i=1}^{l}d(b_{ij}^{\left(t\right)}),\sum_{k=1,k\neq t}^{m}\sum_{j=1}^{n}\sum_{i=1}^{l}d(c_{ij}^{\left(t\right)}))$$ (22)

Where dis(*t*) denotes the total distance of DM *t*, ${\tilde{Z}}_{ijk}$ denotes the TFN converted by DM *k* based on alternative *i* and attribute *j*. To simplify the equations, let *α*^(*t*)^, *β*^(*t*)^ and *γ*^(*t*)^ be the elements of dis(*t*), where

$$\alpha^{\left(t\right)}=\sum_{k=1,k\neq t}^{m}\sum_{j=1}^{n}\sum_{i=1}^{l}d(a_{ij}^{\left(t\right)})$$ (23)

$$\beta^{\left(t\right)}=\sum_{k=1,k\neq t}^{m}\sum_{j=1}^{n}\sum_{i=1}^{l}d(b_{ij}^{\left(t\right)})$$ (24)

$$\gamma^{\left(t\right)}=\sum_{k=1,k\neq t}^{m}\sum_{j=1}^{n}\sum_{i=1}^{l}d(c_{ij}^{\left(t\right)})$$ (25)

The consistency of the decisions made by DM *t* is

$$con(t)={\left(dis(t)\right)}^{-1}=({\left(\alpha^{\left(t\right)}\right)}^{-1},{\left(\beta^{\left(t\right)}\right)}^{-1},{\left(\gamma^{\left(t\right)}\right)}^{-1})$$ (26)

The weight of DM *t* is determined by the equation below.


$$\omega^{\left(t\right)}=\frac{con(t)}{\sum_{t=1}^{m}con(t)}=(\sum_{t=1}^{m}{\left(\alpha^{\left(t\right)}\right)}^{-1},\sum_{t=1}^{m}{\left(\beta^{\left(t\right)}\right)}^{-1},\sum_{t=1}^{m}{\left(\gamma^{\left(t\right)}\right)}^{-1})$$ (27)

Hence, the expert weight of the DMs is denoted as

$$\omega ={\left(\omega^{\left(1\right)},\omega^{\left(2\right)},\ldots ,\omega^{\left(m\right)}\right)}^{T}$$ (28)

**Step 4:** Determine the criteria weight. The criteria weight can be determined by entropy method.

Use the weight average operator to integrate the decisions made by DM *t*:

$$\hat{Z_{ij}}=(\sum_{t=1}^{m}a_{ij}^{\left(t\right)}\omega^{\left(t\right)},\sum_{t=1}^{m}b_{ij}^{\left(t\right)}\omega^{\left(t\right)},\sum_{t=1}^{m}c_{ij}^{\left(t\right)}\omega^{\left(t\right)})$$ (29)

The decision matrix can be denoted as

$$G={\left(g_{ij}\right)}_{l\times n}={\left(\sum_{t=1}^{m}B^{\left(t\right)}\omega \right)}_{l\times n}=\left[\begin{array}{cccc} \left(a_{11}^{\prime },b_{11}^{\prime },c_{11}^{\prime }\right) & \left(a_{12}^{\prime },b_{12}^{\prime },c_{12}^{\prime }\right) & \cdots & \left(a_{1n}^{\prime },b_{1n}^{\prime },c_{1n}^{\prime }\right)\\ \left(a_{21}^{\prime },b_{21}^{\prime },c_{21}^{\prime }\right) & \left(a_{22}^{\prime },b_{22}^{\prime },c_{22}^{\prime }\right) & \cdots & \left(a_{2n}^{\prime },b_{2n}^{\prime },c_{2n}^{\prime }\right)\\ \vdots & \vdots & \ddots & \vdots \\ \left(a_{l1}^{\prime },b_{l1}^{\prime },c_{l1}^{\prime }\right) & \left(a_{l2}^{\prime },b_{l2}^{\prime },c_{l2}^{\prime }\right) & \cdots & \left(a_{ln}^{\prime },b_{ln}^{\prime },c_{ln}^{\prime }\right) \end{array}\right]$$ (30)

Normalize the elements of the decision matrix by the equation below.


$$P_{ij}=(P(a_{ij}),P(b_{ij}),P(c_{ij}),)=\frac{g_{ij}}{\sum_{i=1}^{l}g_{ij}}=\left(\frac{a_{ij}^{\prime }}{\sum_{i=1}^{l}a_{ij}^{\prime }},\frac{b_{ij}^{\prime }}{\sum_{i=1}^{l}b_{ij}^{\prime }},\frac{c_{ij}^{\prime }}{\sum_{i=1}^{l}c_{ij}^{\prime }}\right)$$ (31)

Using the entropy method [59], the measure *e*_*j*_ of each criteria can be determined as

$$e_{j}=-\frac{1}{\ln\left(l\right)}\sum_{i=1}^{l}\;P_{ij}ln(P_{ij})$$ (32)

The criteria weight can be calculated by the following equation.


$$w_{j}=\frac{\left|1-e_{j}\right|}{\sum_{j=1}^{n}\left|1-e_{j}\right|}$$ (33)

**Step 5**: Construct the rough Z-number decision matrix.

For the alternative *i* and attribute *j*, *i* = 1,2,…,*l*, *j* = 1,2,…,*n*, the decisions made by the experts are

$$Z=\{Z_{ij}^{\left(1\right)},Z_{ij}^{\left(2\right)},\ldots ,Z_{ij}^{\left(m\right)}\}$$ (34)

Using the TFNs to illustrate the Z-numbers, the matrix can be converted into

$${\tilde{Z}}_{ij}=\left\{{\tilde{Z}}_{ij}^{\left(1\right)},{\tilde{Z}}_{ij}^{\left(2\right)},\ldots ,{\tilde{Z}}_{ij}^{\left(m\right)}\right\}=\left\{(a_{ij}^{\left(1\right)},b_{ij}^{\left(1\right)},c_{ij}^{\left(1\right)}),(a_{ij}^{\left(2\right)},b_{ij}^{\left(2\right)},c_{ij}^{\left(2\right)}),\ldots ,(a_{ij}^{\left(m\right)},b_{ij}^{\left(m\right)},c_{ij}^{\left(m\right)})\right\}$$ (35)

Afterwards, transform the elements in the matrix into Z-numbers. According to Eq (16), the decisions made by experts in the matrix ${\tilde{Z}}_{ij}$ can be aggregated as a rough Z-number using the equation below.


$$RZ({\tilde{Z}}_{ij})=(\left[a_{ij}^{+},a_{ij}^{-}],[b_{ij}^{+},b_{ij}^{-}],[c_{ij}^{+},c_{ij}^{-}\right])$$ (36)

The rough Z-number decision matrix can be determined as

$$RZ=\left[\begin{array}{cccc} \left(\left[a_{11}^{+},a_{11}^{-}],[b_{11}^{+},b_{11}^{-}],[c_{11}^{+},c_{11}^{-}\right]\right) & \left(\left[a_{12}^{+},a_{12}^{-}],[b_{12}^{+},b_{12}^{-}],[c_{12}^{+},c_{12}^{-}\right]\right) & \cdots & \left(\left[a_{1n}^{+},a_{1n}^{-}],[b_{1n}^{+},b_{1n}^{-}],[c_{1n}^{+},c_{1n}^{-}\right]\right)\\ \left(\left[a_{21}^{+},a_{21}^{-}],[b_{21}^{+},b_{21}^{-}],[c_{21}^{+},c_{21}^{-}\right]\right) & \left(\left[a_{22}^{+},a_{22}^{-}],[b_{22}^{+},b_{22}^{-}],[c_{22}^{+},c_{22}^{-}\right]\right) & \cdots & \left(\left[a_{2n}^{+},a_{2n}^{-}],[b_{2n}^{+},b_{2n}^{-}],[c_{2n}^{+},c_{2n}^{-}\right]\right)\\ \vdots & \vdots & \ddots & \vdots \\ \left(\left[a_{l1}^{+},a_{l1}^{-}],[b_{l1}^{+},b_{l1}^{-}],[c_{l1}^{+},c_{l1}^{-}\right]\right) & \left(\left[a_{l2}^{+},a_{l2}^{-}],[b_{l2}^{+},b_{l2}^{-}],[c_{l2}^{+},c_{l2}^{-}\right]\right) & \cdots & \left(\left[a_{ln}^{+},a_{ln}^{-}],[b_{ln}^{+},b_{ln}^{-}],[c_{ln}^{+},c_{ln}^{-}\right]\right) \end{array}\right]$$ (37)

**Step 6**: Normalize the decision matrix.

The normalized equation can be distinguished by the attribute type [20]. For the Benefit Attributes (BA) such as tensile strength, the elements in the decision matrix can be normalized by Eq (38); for the Cost Attributes (CA) such as total cost, the normalized equation is Eq (39).

For *j*∈*BA*:

$$\hat{RZ}(x_{ij})=\left([\frac{a_{ij}^{-}}{\underset{i}{\max}a_{ij}^{+}},\frac{a_{ij}^{+}}{\underset{i}{\max}a_{ij}^{+}}],[\frac{b_{ij}^{-}}{\underset{i}{\max}b_{ij}^{+}},\frac{b_{ij}^{+}}{\underset{i}{\max}b_{ij}^{+}}],[\frac{c_{ij}^{-}}{\underset{i}{\max}c_{ij}^{+}},\frac{c_{ij}^{+}}{\underset{i}{\max}c_{ij}^{+}}]\right)$$ (38)

For *j*∈*CA*:

$$\hat{RZ}(x_{ij})=\left([\frac{\underset{i}{\min}a_{ij}^{-}}{a_{ij}^{+}},\frac{\underset{i}{\min}a_{ij}^{-}}{a_{ij}^{-}}],[\frac{\underset{i}{\min}b_{ij}^{-}}{b_{ij}^{+}},\frac{\underset{i}{\max}b_{ij}^{+}}{b_{ij}^{-}}],[\frac{\underset{i}{\min}c_{ij}^{-}}{c_{ij}^{+}},\frac{\underset{i}{\min}c_{ij}^{-}}{c_{ij}^{-}}]\right)$$ (39)

Where *i* = 1,2,…,*l*, *j* = 1,2,…,*n*.

**Step 7:** Establish the criteria weighted matrix according to MABAC.


$$WRZ(x_{ij})=[\hat{RZ}(x_{ij})+1]\times w_{j}=([a_{ij}^{*-},a_{ij}^{*+}],[b_{ij}^{*-},b_{ij}^{*+}],[c_{ij}^{*-},c_{ij}^{*+}])$$ (40)

**Step 8**: Build the BAA matrix.

The BAA for each attribute can be calculated using the equation below.


$$r_{i}={\left(\prod_{i=1}^{l}NRZ(x_{ij})\right)}^{\frac{1}{l}}=\left([{\left(\prod_{i=1}^{l}a_{ij}^{*-}\right)}^{\frac{1}{l}},{\left(\prod_{i=1}^{l}a_{ij}^{*+}\right)}^{\frac{1}{l}}],[{\left(\prod_{i=1}^{l}b_{ij}^{*-}\right)}^{\frac{1}{l}},{\left(\prod_{i=1}^{l}b_{ij}^{*+}\right)}^{\frac{1}{l}}],[{\left(\prod_{i=1}^{l}c_{ij}^{*-}\right)}^{\frac{1}{l}},{\left(\prod_{i=1}^{l}c_{ij}^{*+}\right)}^{\frac{1}{l}}]\right)$$ (41)

The BAA matrix can be denoted as

$$R=(r_{1},r_{2},\ldots ,r_{n})$$ (42)

The gap between the alternatives and the BAA can be calculated as

Q=NRZ−R (43)

Which means

$$\left[\begin{array}{cccc} q_{11} & q_{12} & \ldots & q_{1n}\\ q_{21} & q_{22} & & q_{2n}\\ \ldots & \ldots & \ldots & \ldots \\ q_{l1} & q_{l2} & \ldots & q_{ln} \end{array}\right]=\left[\begin{array}{cccc} NRZ(x_{11}) & NRZ(x_{12}) & \ldots & NRZ(x_{1n})\\ NRZ(x_{21}) & NRZ(x_{22}) & & NRZ(x_{2n})\\ \ldots & \ldots & \ldots & \ldots \\ NRZ(x_{l1}) & NRZ(x_{l2}) & \ldots & NRZ(x_{ln}) \end{array}\right]-\left[\begin{array}{c} r_{1}r_{2}\ldots r_{n}\\ r_{1}r_{2}r_{n}\\ \ldots \ldots \ldots \ldots \\ r_{1}r_{2}\ldots r_{n} \end{array}\right]$$ (44)

**Step 9:** Rank the alternatives.

Thus, the values of the attribute functions of alternative *i* can be determined by the equation below.


$$S_{i}=\sum_{j=1}^{n}\;q_{ij}$$ (45)

Where *l* and *n* denote the number of the alternatives and the criteria, respectively, and *i* = 1,2,…,*l*, *j* = 1,2,…,*n*.

The form of *S*_*i*_ can be denoted as

$$S_{i}=\left([{\hat{a}}_{ij}^{+},{\hat{a}}_{ij}^{-}],[{\hat{b}}_{ij}^{+},{\hat{b}}_{ij}^{-}],[{\hat{c}}_{ij}^{+},{\hat{c}}_{ij}^{-}]\right)$$ (46)

Finally, the alternatives can be ranked by the values of the attribute functions. To compare the alternatives, the mean value of *S*_*i*_ is defined. Let $S_{i}^{\prime }$ be the mean value of *S*_*i*_, then

$$S_{i}^{\prime }=\frac{1}{6}({\hat{a}}_{ij}^{+}+{\hat{a}}_{ij}^{-}+{\hat{b}}_{ij}^{+}+{\hat{b}}_{ij}^{-}+{\hat{c}}_{ij}^{+}+{\hat{c}}_{ij}^{-})$$ (47)

If $S_{A}^{\prime }>S_{B}^{\prime }$, alternative *A* is better than alternative *B*. The alternatives can be ranked.

## 5. Case study

**Fig 5:**
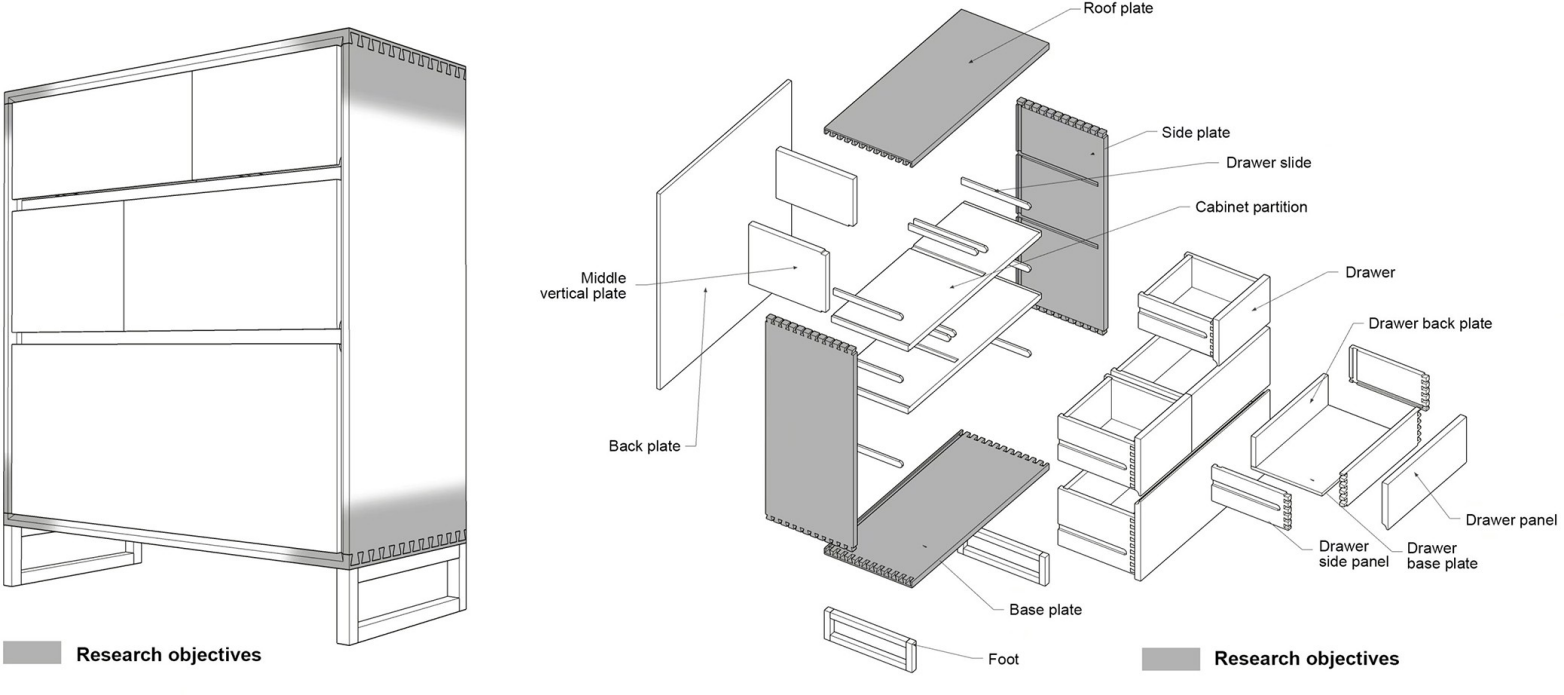
The sketch map of the bucket cabinet.

As is shown in Fig 5, the concept of a store buckets is proposed. The size of the bucket cabinet is 700mm/300mm/820mm, and the bucket cabinet is used for storage, placed indoors against a wall. The material of the cabinet is ash wood. For this work, an appropriate mortise and tenon joint structure must be selected from 60 structures. The details are as follows:

**Phase 1:** Conduct preliminary structure screen by Pugh’s controlled convergence

To reduce the workloads of the experts, over 50 alternatives need to be eliminated in this phase. First, seven criteria are identified: “Bending strength”, “Tensile strength”, “Life duration”, “Production difficulty”, “Process time”, “Cost”, and “Aesthetic”. Then 60 mortise and tenon joint structures are evaluated according to the criteria one by one, using the symbols “+”, “-” and “S”. Then, the total score of each structure can be calculated after converting the symbols into crisp numbers. Finally, the structures with the highest scores can be included in the next phase.

**Fig 6:**
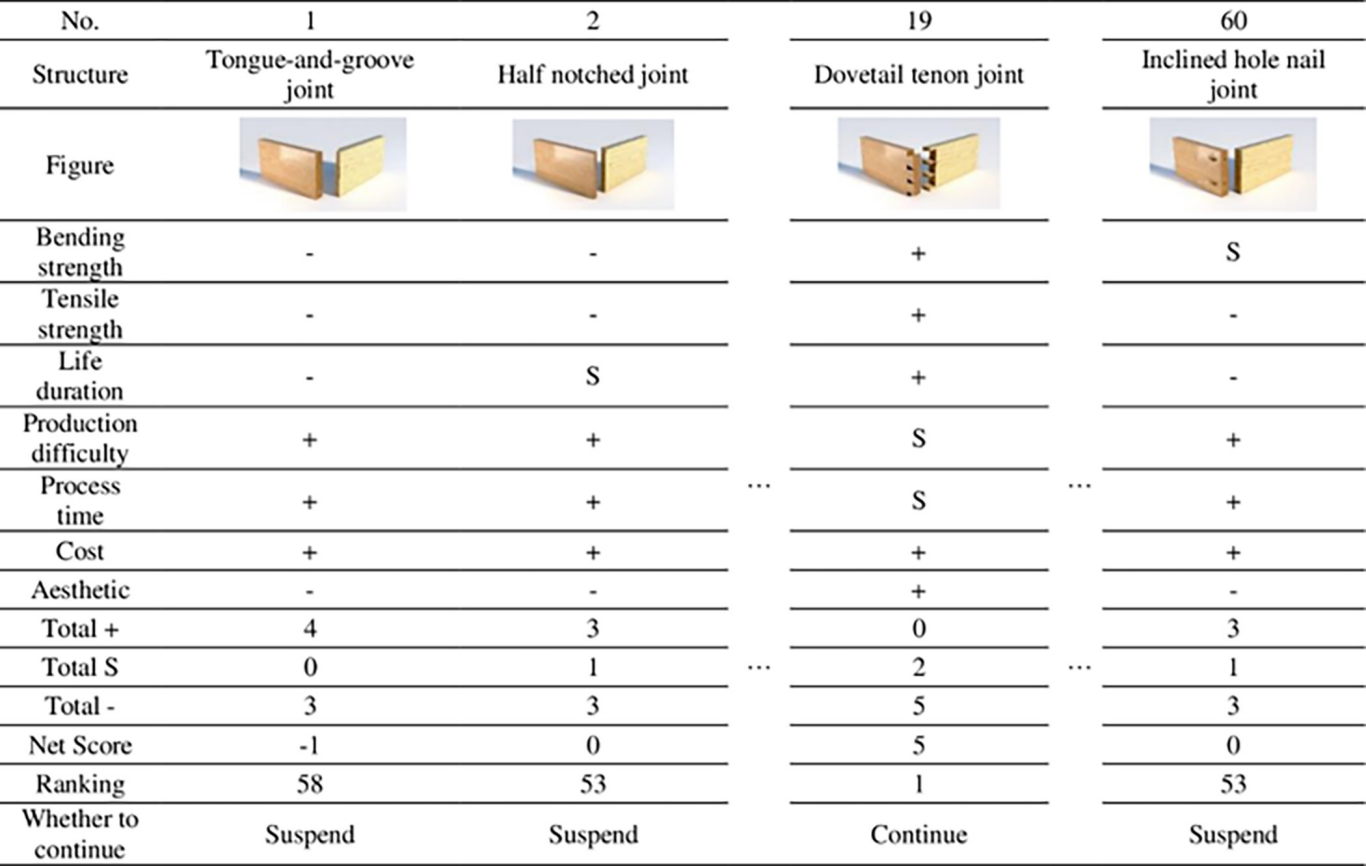
The Pugh decision matrix of the structures.

**Fig 7:**
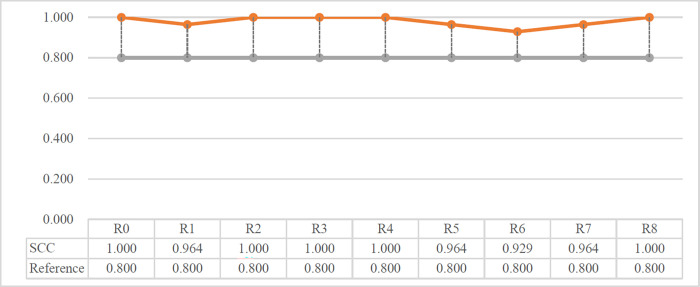
Results of sensitivity analysis.

As is shown in Figs 6, 7 of 60 structures get a score greater than or equal to 5 (the maximum is 7). These 7 structures are selected as the alternatives for the next phase and the other 53 alternatives are eliminated in this phase.

**Phase 2**: Select the optimal mortise and tenon joint by a rough Z-number based MABAC method.

In this phase, 9 experts *E* = {*e*_1_, *e*_2_,…,*e*_9_} are assigned to give their judgements on the 7 alternatives *A* = {*A*_1_, *A*_2_,…,*A*_7_} by linguistic information based on 8 criteria *C* = {*c*_1_, *c*_2_,…,*c*_8_}. Both the restriction information and the reliability are required. The linguistic variants and the criteria are shown in Tables 1 and 2, respectively.

**Table 1:** Linguistic information for the evaluation.

Restriction	Symbol	TFNs	Reliability	Symbol	TFNs
Extremely poor	EP	(0, 0, 1)	Absolutely uncertain	AU	(0, 0, 0.1)
Very poor	VP	(0, 1, 3)	Very uncertain	VU	(0, 0.2, 0.3)
Poor	P	(1, 3, 5)	Uncertain	U	(0.1, 0.3, 0.5)
Fair	F	(3, 5, 7)	Moderate	M	(0.3, 0.5, 0.7)
Good	G	(5, 7, 9)	Certain	C	(0.5, 0.7, 0.9)
Very good	VG	(7, 9, 10)	Very certain	VC	(0.7, 0.8, 1)
Extremely good	EG	(9, 10, 10)	Absolutely certain	AC	(0.9, 1, 1)

**Table 2:** The 8 criteria used in phase 2.

Criteria	Specification	Type	Criteria	Specification	Type
C_1_	Suitable for the product	Benefit	C_5_	Consideration based on technical and craft	Benefit
C_2_	Process time	Benefit	C_6_	Consideration based on structural strength	Benefit
C_3_	Production difficulty	Benefit	C_7_	Aesthetics	Benefit
C_4_	Economical	Benefit	C_8_	Life duration	Benefit

**Step 2.1**: The decision matrix can be built according to the experts’ judgements. The decision matrix of DM *e*_1_ is shown in Eq (48) and transferred into TFNs as shown in Eq (49). Accordingly, the decision matrices of the other DMs can be constructed.


$$A^{\left(1\right)}=\left[\begin{array}{c} \left(\mathrm{VG},\mathrm{VC})(\mathrm{VG},\mathrm{VC})(F,\mathrm{VC})\;(G,\mathrm{VC})(G,\mathrm{VC})(\mathrm{VG},\mathrm{VC})(F,\mathrm{VC})\;(G,\mathrm{VC}\right)\\ \left(\mathrm{VG},\mathrm{VC})\;(G,\mathrm{VC})(G,\mathrm{VC})(G,\mathrm{VC})(F,\mathrm{VC})(\mathrm{EG},\mathrm{VC})(\mathrm{VG},\mathrm{VC})(\mathrm{EG},\mathrm{VC}\right)\\ \left(\mathrm{VG},\mathrm{VC})\;(G,\mathrm{VC})(G,\mathrm{VC})(G,\mathrm{VC})(F,\mathrm{VC})(G,\mathrm{VC})(\mathrm{EG},\mathrm{VC})(F,\mathrm{VC}\right)\\ \left(G,\mathrm{VC})\;(G,\mathrm{VC})(G,\mathrm{VC})(G,\mathrm{VC})(G,\mathrm{VC})\;(P,C)\;(P,\mathrm{VC})\;(F,C\right)\\ \left(F,\mathrm{VC})\;(G,\mathrm{VC})\;(F,\mathrm{VC})(G,\mathrm{VC})(G,\mathrm{VC})\;(P,C)\;(P,C)\;(F,C\right)\\ \left(P,\mathrm{VC})\;\;(P,\mathrm{VC})\;(F,\mathrm{VC})(F,\mathrm{VC})(G,\mathrm{VC})(G,\mathrm{VC})(\mathrm{VG},\mathrm{VC})(\mathrm{VG},\mathrm{VC}\right)\\ \left(\mathrm{EP},\mathrm{VC})\;(G,\mathrm{VC})(G,\mathrm{VC})(G,\mathrm{VC})(G,\mathrm{VC})(\mathrm{VP},\mathrm{VC})\;(F,\mathrm{VC})\;(F,\mathrm{VC}\right) \end{array}\right]$$ (48)

$${\tilde{A}}^{\left(1\right)}=\left[\begin{array}{ccc} \left([7,9,10],[0.7,0.8,1]\right) & \cdots & \left([5,7,9],[0.7,0.8,1]\right)\\ \vdots & \ddots & \vdots \\ \left([0,0,1],[0.7,0.8,1]\right) & \cdots & \left([3,5,7],[0.7,0.8,1]\right) \end{array}\right]$$ (49)

**Step 2.2**: To compare the linguistic information generated by the experts, the Z-numbers matrices are converted into TFN matrices. Take DM *e*_1_ as an example, the decision matrix can be converted as

$$B^{\left(1\right)}=\left[\begin{array}{ccc} \left(6.390,8.216,9.129\right) & \cdots & \left(4.564,6.390,8.216\right)\\ \vdots & \ddots & \vdots \\ \left(0,0,0.913\right) & \cdots & \left(2.739,4.564,6.390\right) \end{array}\right]$$ (50)

**Step 2.3**: Transform every restriction and the corresponding reliability decision made by each DM into a TFN. According to Eq (22), the total distance between DMs can be determined. Based on consistency theory, the consistency of decisions made by each DM can be calculated. Expert weight can be determined by Eq (27). The coefficients and expert weights are shown in Table 3.

**Table 3:** Weight of each expert based on consistency theory.

DM	Total distance (dis(*t*))	Consistency (con(*t*))	Expert weight (*ω*^(*t*)^)
e1	(546.02, 1030.07, 936.59)	(0.00183, 0.00097, 0.00107)	(0.124, 0.119, 0.115)
e2	(642.60, 1162.65, 1050.35)	(0.00156, 0.00086, 0.00095)	(0.105, 0.106, 0.103)
e3	(589.38, 1068.16, 922.59)	(0.00170, 0.00094, 0.00108)	(0.115, 0.115, 0.117)
e4	(657.01, 1158.60, 1026.31)	(0.00152, 0.00086, 0.00097)	(0.103, 0.106, 0.105)
e5	(530.75, 1011.30, 900.99)	(0.00188, 0.00099, 0.00111)	(0.127, 0.122, 0.120)
e6	(567.45, 1051.03, 922.06)	(0.00176, 0.00095, 0.00108)	(0.119, 0.117, 0.117)
e7	(626.67, 1157.52, 993.47)	(0.00160, 0.00086, 0.00101)	(0.108, 0.106, 0.109)
e8	(602.35, 1133.63, 1002.99)	(0.00166, 0.00088, 0.00100)	(0.112, 0.108, 0.108)
e9	(771.93, 1218.67, 1022.80)	(0.00130, 0.00082, 0.00098)	(0.088, 0.101, 0.106)

**Step 2.4**: Determine the weighted decision matrix of each attribute by Eq (30). The weighted matrix of *A*_1_ is shown in Table 4.

**Table 4:** Weighted matrix of alternative *A*_1_.

DM	C_1_	C_2_	C_3_	C_4_
e1	(0.791, 0.980, 1.053)	(0.791, 0.980, 1.053)	(0.339, 0.545, 0.737)	(0.565, 0.762, 0.948)
e2	(0.480, 0.675, 0.845)	(0.096, 0.289, 0.470)	(0.096, 0.289, 0.470)	(0.288, 0.482, 0.657)
e3	(0.732, 0.945, 1.069)	(0.732, 0.945, 1.069)	(0.732, 0.945, 1.069)	(0.732, 0.945, 1.069)
e4	(0.657, 0.871, 0.961)	(0.657, 0.871, 0.961)	(0.845, 0.968, 0.961)	(0.469, 0.678, 0.865)
e5	(0.532, 0.712, 0.903)	(0.532, 0.712, 0.903)	(0.319, 0.508, 0.702)	(0.532, 0.712, 0.903)
e6	(0.498, 0.685, 0.882)	(0.253, 0.413, 0.580)	(0.253, 0.413, 0.580)	(0.498, 0.685, 0.882)
e7	(0.530, 0.731, 0.963)	(0.954, 1.044, 1.069)	(0.954, 1.044, 1.069)	(0.954, 1.044, 1.069)
e8	(0.094, 0.272, 0.451)	(0.094, 0.272, 0.451)	(0.094, 0.272, 0.451)	(0.094, 0.272, 0.451)
e9	(0.431, 0.552, 0.579)	(0.719, 0.921, 0.964)	(0.774, 0.991, 1.039)	(0.659, 0.844, 0.884)
Average	(0.527, 0.714, 0.856)	(0.536, 0.716, 0.836)	(0.490, 0.664, 0.787)	(0.532, 0.714, 0.859)
DM	C_5_	C_6_	C_7_	C_8_
e1	(0.565, 0.762, 0.948)	(0.791, 0.980, 1.053)	(0.339, 0.545, 0.737)	(0.565, 0.762, 0.948)
e2	(0.096, 0.289, 0.470)	(0.480, 0.675, 0.845)	(0.480, 0.675, 0.845)	(0.672, 0.868, 0.939)
e3	(0.732, 0.945, 1.069)	(0.732, 0.945, 1.069)	(0.314, 0.525, 0.748)	(0.732, 0.945, 1.069)
e4	(0.845, 0.968, 0.961)	(0.282, 0.484, 0.673)	(0.282, 0.484, 0.673)	(0.282, 0.484, 0.673)
e5	(0.319, 0.508, 0.702)	(0.532, 0.712, 0.903)	(0.532, 0.712, 0.903)	(0.532, 0.712, 0.903)
e6	(0.498, 0.685, 0.882)	(0.697, 0.880, 0.981)	(0.299, 0.489, 0.686)	(0.498, 0.685, 0.882)
e7	(0.954, 1.044, 1.069)	(0.954, 1.044, 1.069)	(0.742, 0.939, 1.069)	(0.686, 0.751, 0.769)
e8	(0.094, 0.272, 0.451)	(0.469, 0.635, 0.811)	(0.469, 0.635, 0.811)	(0.469, 0.635, 0.811)
e9	(0.659, 0.844, 0.884)	(0.512, 0.759, 0.884)	(0.258, 0.496, 0.727)	(0.240, 0.387, 0.521)
Average	(0.529, 0.702, 0.826)	(0.605, 0.791, 0.921)	(0.413, 0.611, 0.800)	(0.519, 0.692, 0.835)

Using the entropy method, the criteria weight of the 8 criteria can be calculated by Eqs (31) to (33). The criteria weights are shown in Table 5.

**Table 5:** Weighted matrix of alternative *A*_1_.

Criteria	C1	C2	C3	C4
Weight	(0.072, 0.080, 0.080)	(0.092, 0.081, 0.071)	(0.059, 0.050, 0.044)	(0.033, 0.030, 0.027)
Criteria	C5	C6	C7	C8
Weight	(0.065, 0.051, 0.038)	(0.304, 0.301, 0.297)	(0.085, 0.076, 0.077)	(0.290, 0.331, 0.367)

**Step 2.5**: Construct the rough Z-number decision matrix by aggregating the information obtained from the experts. For each alternative and attribute, the judgement can be determined by Eqs (8) to (19). The rough Z-number decision matrix is shown as

$$RZ=\left[\begin{array}{ccc} \left([3.594,5.759],[5.142,7.510],[6.442,8.683]\right) & \cdots & \left([3.719,5.571],[5.228,7.199],[6.581,8.327]\right)\\ \vdots & \ddots & \vdots \\ \left([1.825,4.301],[2.926,5.911],[4.220,7.578]\right) & \cdots & \left([1.513,4.273],[2.635,5.645],[4.003,6.961]\right) \end{array}\right]$$ (51)

**Step 2.6**: Compute the normalized Rough Z-number decision matrix according to Eqs (38) and (39):

$$\hat{RZ}=\left[\begin{array}{ccc} \left([0.103,0.165],[0.111,0.162],[0.115,0.155]\right) & \cdots & \left([0.103,0.154],[0.112,0.154],[0.121,0.153]\right)\\ \vdots & \ddots & \vdots \\ \left([0.052,0.123],[0.063,0.127],[0.075,0.135]\right) & \cdots & \left([0.042,0.118],[0.057,0.121],[0.074,0.128]\right) \end{array}\right]$$ (52)

**Step 2.7**: Determine the weighted rough Z-number decision matrix by Eq (40):

$$WRZ=\left[\begin{array}{ccc} \left([0.127,0.204],[0.137,0.201],[0.140,0.188]\right) & \cdots & \left([0.103,0.154],[0.112,0.154],[0.121,0.153]\right)\\ \vdots & \ddots & \vdots \\ \left([0.064,0.152],[0.078,0.158],[0.091,0.164]\right) & \cdots & \left([0.082,0.230],[0.113,0.242],[0.147,0.256]\right) \end{array}\right]$$ (53)

**Step 2.8**: Calculate the BAA of the weighted decision, and compute the gap matrix *Q* between the alternatives and the BAAs by Eqs (41) to (44):

$$Q=\left[\begin{array}{ccc} \left(\left[0.029,0.037],[0.024,0.027],[0.015,0.019\right]\right) & \cdots & \left(\left[0.036,0.066],[0.030,0.058],[0.023,0.047\right]\right)\\ \vdots & \ddots & \vdots \\ \left(\left[-0.026,-0.023],[-0.032,-0.018],[-0.030,-0.009\right]\right) & \cdots & \left(\left[-0.053,-0.034],[-0.053,-0.036],[-0.047,-0.027\right]\right) \end{array}\right]$$ (54)

**Step 2.9**: Finally, calculate the values of the attribute functions according to Eqs (45) to (47), as shown in Table 6. By comparing the average of the values, the optimal alternative can be selected. In this case the “Dovetail tenon joint” is then selected as the optimal option.

**Table 6:** The rankings of the 7 alternatives according to MABAC.

Alternative	Values of the attribute functions	Average	Ranking
A1	([0.223,0.338],[0.170,0.222],[0.117,0.125])	0.199	2
A2	([0.266,0.298],[0.201,0.208],[0.100,0.153])	0.204	1
A3	([0.124,0.132],[0.087,0.122],[0.051,0.114])	0.105	3
A4	([-0.182,-0.083],[-0.148,-0.086],[-0.096,-0.078])	-0.112	7
A5	([-0.140,-0.075],[-0.113,-0.083],[-0.077,-0.072])	-0.093	6
A6	([-0.028,0.003],[-0.017,-0.008],[-0.014,0.006])	-0.012	4
A7	([-0.132,-0.069],[-0.104,-0.102],[-0.099,-0.052])	-0.093	5

## 6. Sensitivity analysis, comparison and discussion

### 6.1 Sensitivity analysis

**Fig 8:**
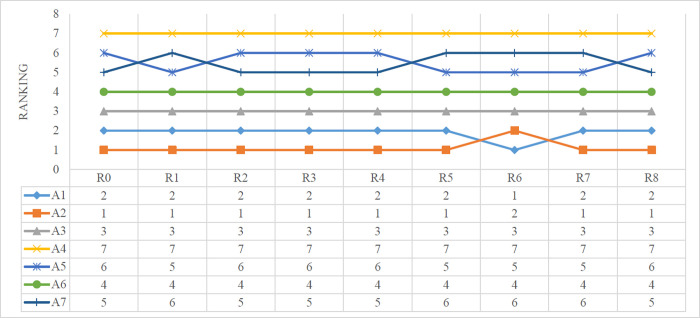
The SCC for weight change experiment.

A sensitivity analysis is conducted to investigate the robustness of the proposed method. Firstly, the criteria weight change experiment is introduced to show the sensitivity of the proposed rough Z-number MABAC method. Figs 7 and 8 show the different increased levels of the most significant attribute selected. In the initial round R0, each attribute remains at the normal criteria weight. In round R1 to round R8, the weight of the corresponding attribute C1 to C8 was increased to 1.8 times the original value. For example, in R1, the attribute weight of C1 is enlarged by 80%, while weights of the other attributes and other variants remain the original values, the rankings of the alternatives of R1 are calculated, and so do the other rounds. We can infer from Fig 7, the rankings of the alternatives have a high degree of consistency. A2 gets 7 first places out of 8 rounds, except a second place in R6. The alternatives A3, A4 and A6 keep the same ranking all through the experiment, only A5 and A7 represent ups and downs in comparison. This is also confirmed by Spearman’s coefficient correlation (SCC) which is shown in Fig 8. High consistency causes Spearman’s coefficients well over 0.8, to stay at the interval [0.9,1]. The sensitivity analysis reveals a high robustness of the proposed method. The ranking order of the alternatives basically remains in a stable state while the criteria weights have large fluctuations.

### 6.2 Comparison and discussion

Comparative analyses are made to identify the effectiveness and efficiency of the proposed method in Phase 2.

**Fig 9:**
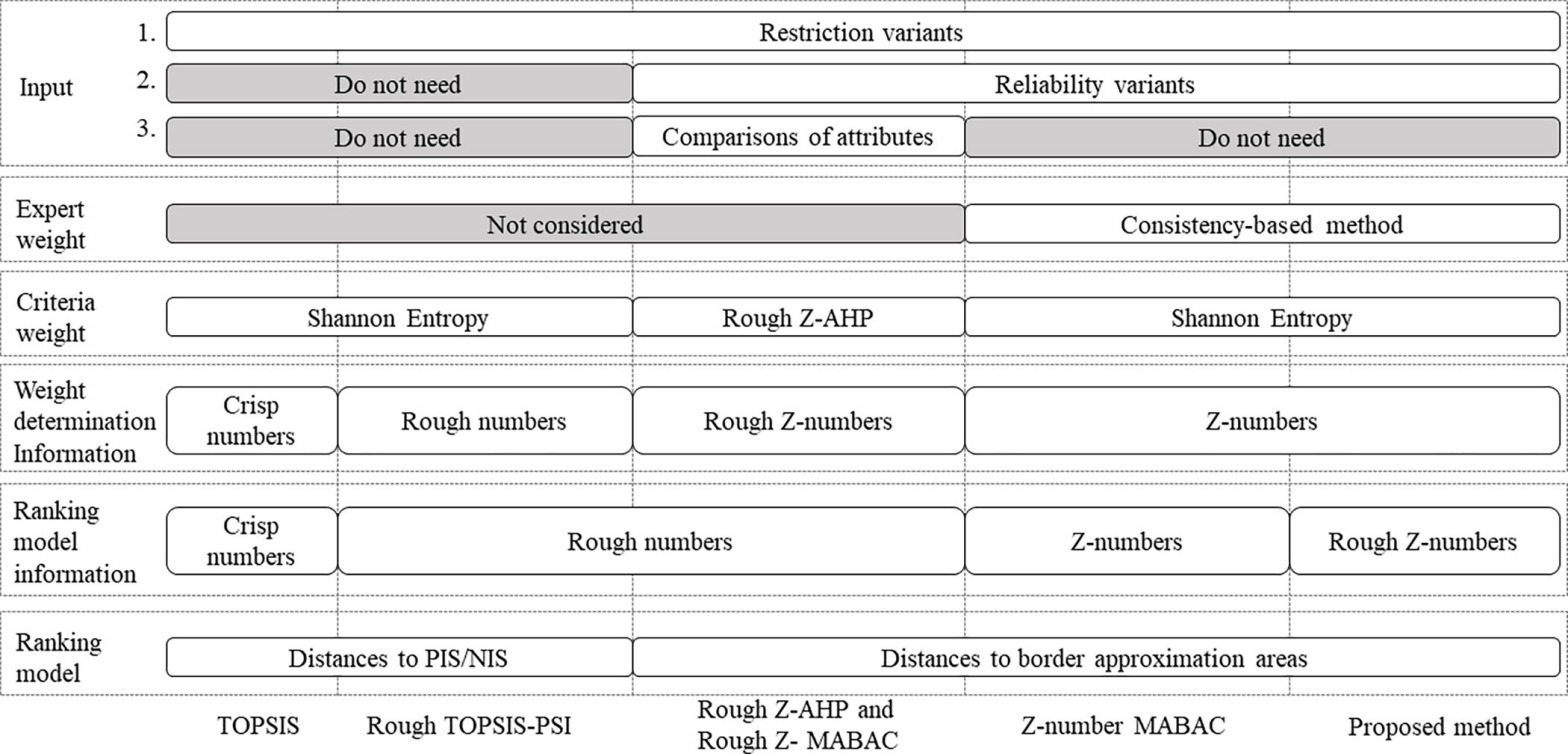
The difference among some MADM methods.

First, as shown in Fig 9, we compared the processes of the TOPSIS method [60], the Rough TOPSIS-PSI method [20], the Rough Z-AHP and Rough Z-MABAC method [56], Z-number MABAC and the proposed method in our study.

1. The input information of the methods is compared. TOPSIS method and Rough TOPSIS-PSI do not need additional information except for the preferences of the alternatives (restriction variants). As an effective way to illustrate the reliability of the experts, both the restriction and the reliability variants are essential for Z-number based methods in the decision-making process [36]. Moreover, the Rough Z-AHP and Rough Z-MABAC methods utilize a Rough Z-AHP method to determine the criteria weight, additional pairwise comparison information among the experts is required as the input for criteria weight determination. However, the selected experts are not always familiar with each other, which means pairwise comparison information is not always available for evaluation.
2. The weight determination methods are compared. The criteria weights are calculated by an objective entropy method in the TOPSIS method, Rough TOPSIS-PSI method, Z-number MABAC method and the proposed method, while Rough Z-AHP and Rough Z- MABAC used a Z-number enhanced AHP method to determine the criteria weight. Moreover, in the Z-number MABAC method and the proposed method, the expert weight is considered and determined by a consistency-based method.
3. The data environments are compared. The uncertain and imprecise structure selection can be divided into reliability characterization, uncertain representation and subjectivity manipulation. The rough set is mainly for subjectivity manipulation while Z-number concentrates on reliability characterization and uncertain representation. As is shown in Fig 9, the data environments include crisp number, rough number, Z-number and rough Z-number.
4. The ranking models are compared. The methods are based on the TOPSIS method and the MABAC method. The TOPSIS compared the alternatives by the distance between themselves and the Positive Ideal Solution (PIS) and the Negative Ideal Solution (NIS), and the MABAC method compared the alternatives through BAA and the values of the attribute functions.

**Fig 10:**
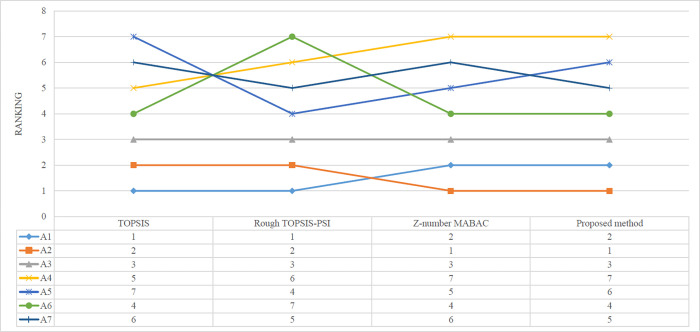
The rankings of the alternatives determined by different methods.

The rankings of the alternatives are also calculated by the compared methods. The Rough Z-AHP and Rough Z-MABAC are not included because the raw data of the rough Z-number enhanced AHP weight determination method is not available. As is shown in Fig 10 the rankings are ordered as A1≻A2≻A3≻A5≻A7≻A4≻A6 in TOPSIS and A1≻A2≻A3≻A6≻A4≻A7≻A5 in Rough TOPSIS-PSI methods, showing A1 is the optimal alternative. By contrast, the rankings are ordered as A2≻A1≻A3≻A7≻A5≻A4≻A6 in both Z-number MABAC and the proposed method, showing A2 is the selected structure.

**Fig 11:**
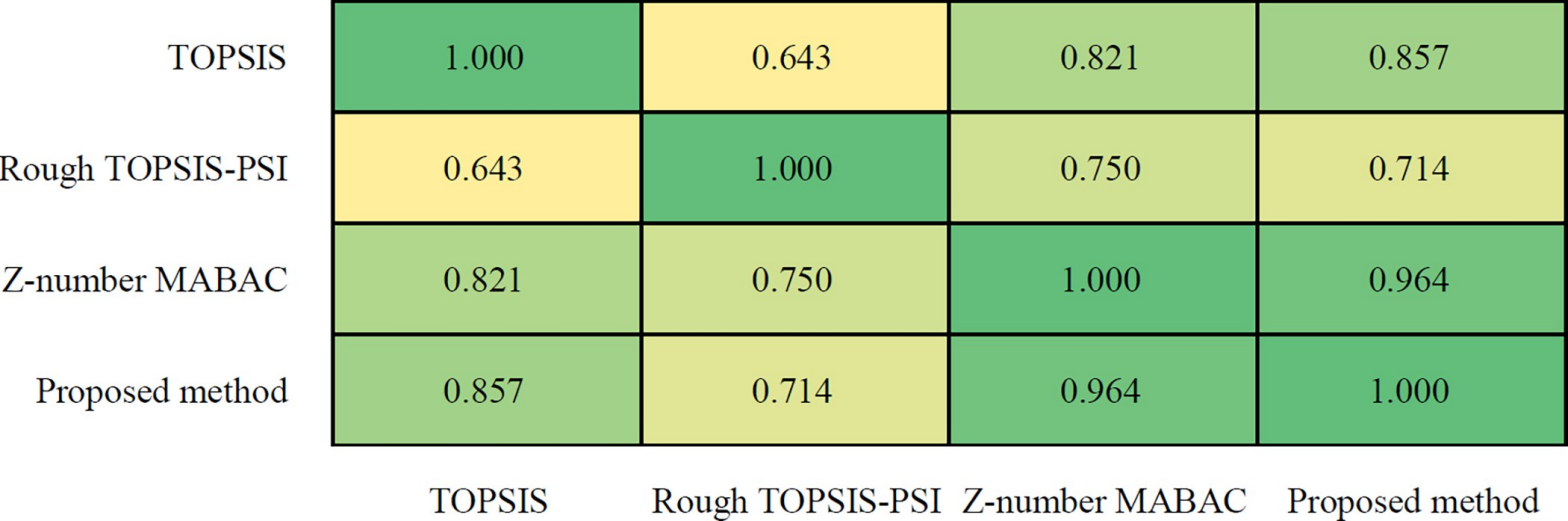
The Spearman correlation coefficients between the results of the methods.

The SCCs among the results of the methods are shown in Fig 11. It is obvious that the correlation between the TOPSIS, the Rough TOPSIS-PSI and the Z-number based method is not very high, because neither the TOPSIS method nor the Rough TOPSIS-PSI method considered the reliability in the evaluation. The figure also shows that the correlation between the Z-number MABAC and the proposed Rough Z-number MABAC is very high, up to 0.964. However, the ranking orders between these two methods are not the same, which identifies the role of the rough set.

## 7. Conclusions

The MABAC method is proven as an excellent MADM method in multiple fields [33,55,61,62]. However, the reliability and uncertain and subjective of the experts’ information and the workload for massive alternatives pose a challenge in the decision-making process. To solve the problem, this paper introduced a comprehensive structure selection method with two phases. In Phase 1, Pugh’s controlled convergence method is used to eliminate unsuitable methods. Most of the alternatives are weeded out in this phase, and only a small proportion of the structures are included in the next phase. In Phase 2, a MABAC method based on a rough Z-number is proposed. First, uncertain Z-numbers are converted into TFNs. Then, the distance determination method and consistency-based theory are aggregated to determine the expert weight. Then, the criteria weight is determined by the entropy method based on the weighted matrix. The decision matrix is integrated and transformed into a rough Z-number matrix. The alternatives are then ranked by comparing the values of the attribute functions. Finally, the application of the proposed method is illustrated. Analysis studies were conducted to reveal the efficiency and effectiveness of the proposed method.

The novelty of this study reveals three aspects. First, an improved rough Z-number MABAC method is proposed in our study. Decision-making approaches and methods such as rough number, Z-number, consistency theory and Shannon entropy are utilized for a MABAC method based model for product structure selection. Second, the uncertain, reliable and subjective information obtained from the experts are considered, and Z-number and rough number are aggregated to solve it in decision-making. Third, to make the decision-making process more efficient, Pugh’s controlled convergence method is applied as the preliminary phase based on a large number of alternatives. As an effective method for selecting a solution from many alternatives, the proposed method can be applied in various areas for more complex decision-making problems.

Although in this paper an innovative mortise and tenon structure selection method for many alternatives is proposed, there are still some limitations. We assume there is not any nonlinear correlation between indexes, because the correlation between indexes may cause imprecise evaluation. However, in real-life cases, it is unable to remove nonlinear correlation between indexes.

In our study, the information obtained is converted into Z-number, computed based on the rough number and TFN. In future studies, the relationship between linguistic information and fuzzy numbers needs to be further clarified, and more mapping functions and calculating methods need to be developed to improve the precision of the decision-making process.

## Supporting information

S1 Appendix PUGH decision matrix of the mortise and tenon joint structures. (PDF)

S2 Appendix Decisions made by the experts. (PDF)

## References

[1] Qiao W, Wang Z, Wang D, Zhang L. A new mortise and tenon timber structure and its automatic construction system. Journal of Building Engineering. 2022;44. doi: 10.1016/j.jobe.2021.103369 WOS:000706980100002.

[2] Wang Z, Song P, Pauly M. MOCCA: Modeling and Optimizing Cone-joints for Complex Assemblies. ACM Transactions on Graphics. 2021;40(4). doi: 10.1145/3450626.3459680 WOS:000674930900145.

[3] He J, Yu P, Wang J, Yang Q, Han M, Xie L. Theoretical model of bending moment for the penetrated mortise-tenon joint involving gaps in traditional timber structure. Journal of Building Engineering. 2021;42. doi: 10.1016/j.jobe.2021.103102 WOS:000689337400005.

[4] Weili L. Application of mortise and tenon structure in modern furniture design (in Chinese). Design. 2020;33(08):102–4.

[5] Xie Q, Zhang B, Li S, Wu F, Yang H. Effects of timber infill walls on the seismic behavior of traditional Chinese timber frames. Earthquake Engineering and Engineering Vibration. 2022;21(4):999–1018. doi: 10.1007/s11803-022-2132-1 WOS:000886991400009.

[6] Zhang B, Xie Q, Liu Y, Zhang L, Li S. Effects of gaps on the seismic performance of traditional timber frames with straight mortise-tenon joint: Experimental tests, energy dissipation mechanism and hysteretic model. Journal of Building Engineering. 2022;58. doi: 10.1016/j.jobe.2022.105019 WOS:000855014500001.

[7] Aydogan S, Gunay EE, Akay D, Kremer GEO. Concept design evaluation by using Z-axiomatic design. Computers in Industry. 2020;122. doi: 10.1016/j.compind.2020.103278 WOS:000570127300001.

[8] Chen Z, Zhong P, Liu M, Ma Q, Si G. A novel integrated MADM method for design concept evaluation. Scientific Reports. 2022;12(1):15885. doi: 10.1038/s41598-022-20044-7 MEDLINE:36151244. 36151244 PMC9508270

[9] Peng H-g, Wang X-k, Wang J-q. New MULTIMOORA and pairwise evaluation-based MCDM methods for hotel selection based on the projection measure of Z-numbers. International Journal of Fuzzy Systems. 2022;24(1):371–90. doi: 10.1007/s40815-021-01141-7 WOS:000678271100002.

[10] Jafarzadeh H, Heidary-Dahooie J, Akbari P, Qorbani A. A project prioritization approach considering uncertainty, reliability, criteria prioritization, and robustness. Decision Support Systems. 2022;156. doi: 10.1016/j.dss.2022.113731 WOS:000820515200001.

[11] Wang Z, Fung RY, Li Y-L, Pu Y. An integrated decision-making approach for designing and selecting product concepts based on QFD and cumulative prospect theory. International Journal of Production Research. 2018;56(5):2003–18.

[12] Shinno H, Yoshioka H, Marpaung S, Hachiga S. Quantitative SWOT analysis on global competitiveness of machine tool industry. Journal of Engineering Design. 2006;17(03):251–8.

[13] Boghani HC, Ambur R, Blumenfeld M, Saade L, Goodall RM, Ward CP, et al. Sensitivity enriched multi-criterion decision making process for novel railway switches and crossings—a case study. European Transport Research Review. 2021;13(1). doi: 10.1186/s12544-020-00467-x WOS:000609270800001.

[14] Ulrich K, Eppinger S. Product design and manufacturing. The McGraw-Hill Inc; 2000.

[15] Bozorg-Haddad O, Zolghadr-Asli B, Loaiciga HA. A handbook on multi-attribute decision-making methods: John Wiley & Sons; 2021.

[16] Ocampo L. Full consistency method (FUCOM) and weighted sum under fuzzy information for evaluating the sustainability of farm tourism sites. Soft Computing. 2022;26(22):12481–508. doi: 10.1007/s00500-022-07184-8 WOS:000805893500004.

[17] Zhu G, Hu J, Ren H. A fuzzy rough number-based AHP-TOPSIS for design concept evaluation under uncertain environments. Applied Soft Computing. 2020;91:106228.

[18] Liu Y, Eckert CM, Earl C. A review of fuzzy AHP methods for decision-making with subjective judgements. Expert Systems with Applications. 2020;161:113738.

[19] Yazdani M, Payam AF. A comparative study on material selection of microelectromechanical systems electrostatic actuators using Ashby, VIKOR and TOPSIS. Materials & Design (1980–2015). 2015;65:328–34.

[20] Chen Z, Zhong P, Liu M, Sun H, Shang K. A novel hybrid approach for product concept evaluation based on rough numbers, shannon entropy and TOPSIS-PSI. Journal of Intelligent & Fuzzy Systems. 2021;40(6):12087–99. doi: 10.3233/jifs-210184 WOS:000667508800118.

[21] Stević Ž, Pamučar D, Puška A, Chatterjee P. Sustainable supplier selection in healthcare industries using a new MCDM method: Measurement of alternatives and ranking according to COmpromise solution (MARCOS). Computers & Industrial Engineering. 2020;140:106231.

[22] Ul Haq RS, Saeed M, Mateen N, Siddiqui F, Naqvi M, Yi JB, et al. Sustainable material selection with crisp and ambiguous data using single-valued neutrosophic-MEREC-MARCOS framework. Applied Soft Computing. 2022;128:109546. 10.1016/j.asoc.2022.109546.

[23] Zhou L-P, Wan S-P, Dong J-Y. A Fermatean fuzzy ELECTRE method for multi-criteria group decision-making. Informatica. 2022;33(1):181–224. doi: 10.15388/21-infor463 WOS:000766621900008.

[24] Molla MU, Giri BC, Biswas P. Extended PROMETHEE method with Pythagorean fuzzy sets for medical diagnosis problems. Soft Computing. 2021;25(6):4503–12. doi: 10.1007/s00500-020-05458-7 WOS:000607092300001.

[25] Saha A, Reddy J, Kumar R. A Fuzzy Similarity Based Classification with Archimedean-Dombi Aggregation Operator. Journal of Intelligent Management Decision. 2022;1(2):118–27.

[26] Kabak Ö, Ervural B. Multiple attribute group decision making: A generic conceptual framework and a classification scheme. Knowledge-Based Systems. 2017;123:13–30.

[27] Lipušček I, Bohanec M, Oblak L, Zadnik Stirn L. A multi-criteria decision-making model for classifying wood products with respect to their impact on environment. The International Journal of Life Cycle Assessment. 2010;15(4):359–67. doi: 10.1007/s11367-010-0157-6

[28] Sibagariang R, Riandari F. Decision Support System for Determining the Best Wood For the Production Cabinet Using Bayes Method: Decision Support System for Determining the Best Wood For the Production Cabinet Using Bayes Method. Jurnal Mantik. 2019;3(3):99–103.

[29] Peng C, Feng D, Guo S. Material Selection in Green Design: A Method Combining DEA and TOPSIS. Sustainability. 2021;13(10). doi: 10.3390/su13105497 WOS:000662479100001.

[30] Cui X, Ge M, Shen X. Application of Comprehensive Evaluation in New-Product-Development Evaluation: The Case of Landscape-Architectural Outdoor Wooden Furnishing. Forests. 2022;13(10). doi: 10.3390/f13101552 WOS:000875021300001.

[31] Zhai L-Y, Khoo L-P, Zhong Z-W. A rough set enhanced fuzzy approach to quality function deployment. International Journal of Advanced Manufacturing Technology. 2008;37(5–6):613–24.

[32] Qi J, Hu J, Peng Y. Modified rough VIKOR based design concept evaluation method compatible with objective design and subjective preference factors. Applied Soft Computing. 2021;107:107414.

[33] Huang G, Li M, Pedrycz W, Pamucar D, Zhang G, Martinez L. Design alternative assessment and selection: A novel Z-cloud rough number-based BWM-MABAC model. Information Sciences. 2022;603:149–89. doi: 10.1016/j.ins.2022.04.040

[34] Wang T-C, Lee H-D. Developing a fuzzy TOPSIS approach based on subjective weights and objective weights. Expert Systems with Applications. 2009;36(5):8980–5.

[35] Hayat K, Ali MI, Karaaslan F, Cao B-Y, Shah MH. Design concept evaluation using soft sets based on acceptable and satisfactory levels: an integrated TOPSIS and Shannon entropy. Soft Computing. 2020;24(3):2229–63. doi: 10.1007/s00500-019-04055-7 WOS:000518595800041.

[36] Qi J, Hu J, Huang H, Peng Y. New customer-oriented design concept evaluation by using improved Z-number-based multi-criteria decision-making method. Advanced Engineering Informatics. 2022;53. doi: 10.1016/j.aei.2022.101683 WOS:000825367100003.

[37] Pugh S. Total design: integrated methods for successful product engineering. 1991.

[38] Mia M, Gupta MK, Singh G, Krolczyk G, Pimenov DY. An approach to cleaner production for machining hardened steel using different cooling-lubrication conditions. Journal of Cleaner Production. 2018;187:1069–81. doi: 10.1016/j.jclepro.2018.03.279 WOS:000432102500093.

[39] Kim M-G, Kim S-W. Impact localization for composite plate using the modified error-outlier algorithm with Pugh’s concept selection under various temperatures. Composite Structures. 2021;272. doi: 10.1016/j.compstruct.2021.114226 WOS:000679385100003.

[40] Li Y, Ghazilla RAR, Abdul-Rashid SH. QFD-based research on sustainable user experience optimization design of smart home products for the elderly: A case study of smart refrigerators. International Journal of Environmental Research and Public Health. 2022;19(21). doi: 10.3390/ijerph192113742 WOS:000881067600001. 36360620 PMC9654730

[41] Zhu T-L, Li Y-J, Wu C-J, Yue H, Zhao Y-Q. Research on the design of surgical auxiliary equipment based on AHP, QFD, and PUGH decision matrix. Mathematical Problems in Engineering. 2022;2022. doi: 10.1155/2022/4327390 WOS:000877882500003.

[42] Lennon E, Farr J, Sesser R. Evaluation of multi-attribute decision making systems applied during the concept design of new microplasma devices. Expert Systems with Applications. 2013;40(16):6321–9. doi: 10.1016/j.eswa.2013.05.049 WOS:000322857200011.

[43] Li Y, Rao C, Goh M, Xiao X. Novel multi-attribute decision-making method based on Z-number grey relational degree. Soft Computing. 2022;26(24):13333–47. doi: 10.1007/s00500-022-07487-w WOS:000860381600001.

[44] Banerjee R, Pal SK. Z*-numbers: Augmented Z-numbers for machine-subjectivity representation. Information Sciences. 2015;323:143–78.

[45] Allahviranloo T, Ezadi S. Z-Advanced numbers processes. Information Sciences. 2019;480:130–43.

[46] Ahmadov SA, editor Z+-number based alternatives selection in investment problem. International Conference on Theory and Application of Soft Computing, Computing with Words and Perceptions; 2021: Springer.

[47] Zadeh LA. A note on Z-numbers. Information Sciences. 2011;181(14):2923–32.

[48] Stević Ž, Zavadskas EK, Tawfiq FM, Tchier F, Davidov T. Fuzzy Multicriteria Decision-Making Model Based on Z Numbers for the Evaluation of Information Technology for Order Picking in Warehouses. Applied Sciences. 2022;12(24):12533.

[49] Kang B, Wei D, Li Y, Deng Y. A method of converting Z-number to classical fuzzy number. Journal of Information &computational Science. 2012;9(3):703–9.

[50] Božanić D, Jurišić D, Erkić D. LBWA–Z-MAIRCA model supporting decision making in the army. Operational Research in Engineering Sciences: Theory and Applications. 2020;3(2):87–110.

[51] Garg A, Maiti J, Kumar A. Granulized Z‐OWA aggregation operator and its application in fuzzy risk assessment. International Journal of Intelligent Systems. 2022;37(2):1479–508.

[52] Cheng R, Zhang J, Kang B. A novel Z-TOPSIS method based on improved distance measure of Z-numbers. International Journal of Fuzzy Systems. 2022;24(6):2813–30. doi: 10.1007/s40815-022-01297-w WOS:000800991200001.

[53] Jia Q, Hu J, He Q, Zhang W, Safwat E. A multicriteria group decision-making method based on AIVIFSs, Z-numbers, and trapezium clouds. Information Sciences. 2021;566:38–56. doi: 10.1016/j.ins.2021.02.042 WOS:000656939300003.

[54] Tian Z-p, Nie R-x, Wang J-q, Luo H, Li. A prospect theory-based QUALIFLEX for uncertain linguistic Z-number multi-criteria decision-making with unknown weight information. Journal of Intelligent & Fuzzy Systems. 2020;38(2):1775–87. doi: 10.3233/jifs-190065 WOS:000514081100064.

[55] Shen K-w Wang X-k, Qiao D Wang J-q. Extended Z-MABAC method based on regret theory and directed distance for regional circular economy development program selection with Z-information. Ieee Transactions on Fuzzy Systems. 2020;28(8):1851–63. doi: 10.1109/tfuzz.2019.2923948 WOS:000557355500028.

[56] Zhu G-N. Design concept evaluation considering information reliability, uncertainty, and subjectivity: An integrated rough-Z-number-enhanced MCGDM methodology. Advanced Engineering Informatics. 2022;54:101796. 10.1016/j.aei.2022.101796.

[57] Sadi-Nezhad S, Noroozi-yadak A, Makui A. Fuzzy distance of triangular fuzzy numbers. Journal of Intelligent & Fuzzy Systems. 2013;25(4):845–52.

[58] Chen Z, Zhong P, Liu M, Ma Q, Si G. An integrated expert weight determination method for design concept evaluation. Scientific Reports. 2022;12(1):6358. doi: 10.1038/s41598-022-10333-6 35428829 PMC9012764

[59] Chatterjee K, Kar S. A multi-criteria decision making for renewable energy selection using Z-numbers in uncertain environment. Technological and Economic Development of Economy. 2018;24(2):739–64. doi: 10.3846/20294913.2016.1261375 WOS:000429149800021.

[60] Behzadian M, Otaghsara SK, Yazdani M, Ignatius J. A state-of the-art survey of TOPSIS applications. Expert Systems with Applications. 2012;39(17):13051–69.

[61] Pamučar D, Stević Ž, Zavadskas EK. Integration of interval rough AHP and interval rough MABAC methods for evaluating university web pages. Applied soft computing. 2018;67:141–63.

[62] Pamučar D, Ćirović G. The selection of transport and handling resources in logistics centers using Multi-Attributive Border Approximation area Comparison (MABAC). Expert systems with applications. 2015;42(6):3016–28.

