# Supplementary material for: A comprehensive mortise and tenon structure selection method based on Pugh’s controlled convergence and rough Z-number MABAC method
Source: PLoS One. 2023 May 18;18(5):e0283704. doi: 10.1371/journal.pone.0283704 (PMC10194880; doi:10.1371/journal.pone.0283704)
Supplement: S1 Appendix — (PDF) [file pone.0283704.s001.pdf]

Appendix 1: PUGH decision matrix of the mortise and tenon joint structures

| No. | Mortise and tenon joint structure ( Board Corner Joint) | Figure | Bending strength | Tensile strength | Life duration | Production difficulty | Process time | Cost | Aesthetic | Total - | Total S | Total + | Net Score | Ranking | Weather to continue |
|---|---|---|---|---|---|---|---|---|---|---|---|---|---|---|---|
| 1 | Tongue-and-groove joint | 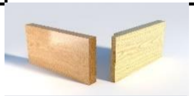 | - | - | - | + | + | + | - | 4 | 0 | 3 | -1 | 58 | Suspend |
| 2 | Half notched joint | 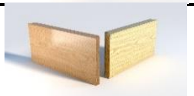 | - | - | S | + | + | + | - | 3 | 1 | 3 | 0 | 53 | Suspend |
| 3 | 45 °half notch joint | 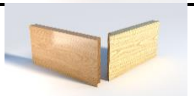 | - | - | S | + | + | S | S | 2 | 3 | 2 | 0 | 53 | Suspend |
| 4 | Concave-convex joint | 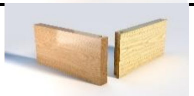 | + | + | + | + | + | S | - | 1 | 1 | 5 | 4 | 8 | Suspend |
| 5 | 45 °concave-convex joint | 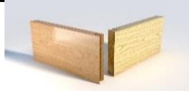 | S | + | + | + | + | S | S | 0 | 3 | 4 | 4 | 8 | Suspend |
| 6 | 45 °half concave-convex joint | 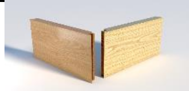 | S | + | + | + | S | S | S | 0 | 4 | 3 | 3 | 24 | Suspend |
| 7 | Half side sharp corner joint | 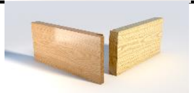 | + | S | S | + | + | S | - | 1 | 3 | 3 | 2 | 32 | Suspend |
| 8 | Half length lap joint | 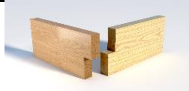 | - | - | - | + | + | + | - | 4 | 0 | 3 | -1 | 58 | Suspend |
| 9 | Half length & thickness lap joint | 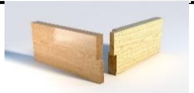 | - | - | - | + | + | + | - | 4 | 0 | 3 | -1 | 58 | Suspend |
| 10 | Trisection joint | 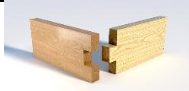 | S | S | - | + | + | + | - | 2 | 2 | 3 | 1 | 38 | Suspend |
| 11 | Hook tenon joint | 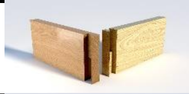 | + | + | S | - | - | S | + | 2 | 2 | 3 | 1 | 38 | Suspend |

| | | | | | | | | | | | | | | | |
|---|---|---|---|---|---|---|---|---|---|---|---|---|---|---|---|
| 12 | Straight mortise joint | 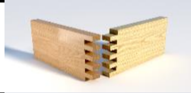 | + | + | + | + | + | S | S | 0 | 2 | 5 | 5 | 1 | Continue |
| 13 | Half side straight mortise joint | 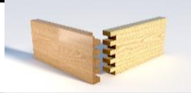 | + | + | + | S | S | S | S | 0 | 4 | 3 | 3 | 24 | Suspend |
| 14 | Comb tenon joint | 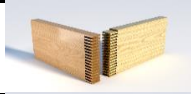 | + | + | + | S | S | S | + | 0 | 3 | 4 | 4 | 8 | Suspend |
| 15 | Oblique comb tenon joint | 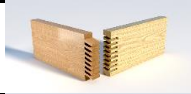 | + | + | + | - | - | - | + | 3 | 0 | 4 | 1 | 38 | Suspend |
| 16 | Intermittent comb tenon joint | 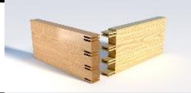 | + | + | + | S | S | S | + | 0 | 3 | 4 | 4 | 8 | Suspend |
| 17 | 45 °edge straight mortise joint | 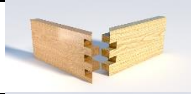 | + | + | + | S | S | + | S | 0 | 3 | 4 | 4 | 8 | Suspend |
| 18 | Straight mortise joint | 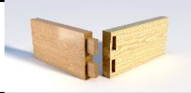 | + | + | + | + | - | + | S | 1 | 1 | 5 | 4 | 8 | Suspend |
| 19 | Dovetail tenon joint | 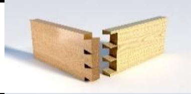 | + | + | + | S | S | + | + | 0 | 2 | 5 | 5 | 1 | Continue |
| 20 | 45 °edge dovetail tenon joint | 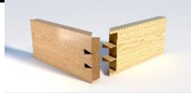 | + | + | + | S | S | S | + | 0 | 3 | 4 | 4 | 8 | Suspend |
| 21 | Half side dovetail tenon joint | 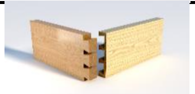 | + | + | + | S | S | + | + | 0 | 2 | 5 | 5 | 1 | Continue |
| 22 | Half side hidden dovetail tenon joint | 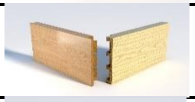 | + | + | + | - | - | S | S | 2 | 2 | 3 | 1 | 38 | Suspend |
| 23 | Hidden dovetail tenon joint | 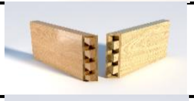 | + | + | + | - | - | - | + | 3 | 0 | 4 | 1 | 38 | Suspend |
| 24 | Straight edge hidden dovetail tenon joint | 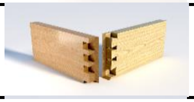 | + | + | + | - | - | - | + | 3 | 0 | 4 | 1 | 38 | Suspend |

| | | | | | | | | | | | | | | |
|---|---|---|---|---|---|---|---|---|---|---|---|---|---|---|
| 25 | Arcuate dovetail tenon joint | 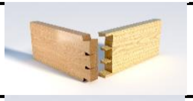 | + | + | + | - | - | - | + | 3 | 0 | 4 | 1 | 38 | Suspend |
| 26 | Comb dovetail tenon joint | 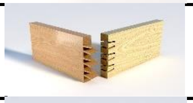 | + | + | + | - | - | - | + | 3 | 0 | 4 | 1 | 38 | Suspend |
| 27 | Oblique comb dovetail tenon joint | 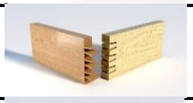 | + | + | + | - | - | - | + | 3 | 0 | 4 | 1 | 38 | Suspend |
| 28 | Slotted dovetail tenon joint | 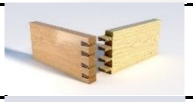 | + | + | + | - | - | - | + | 3 | 0 | 4 | 1 | 38 | Suspend |
| 29 | Trapezoidal dovetail tenon joint | 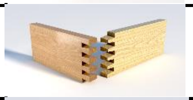 | + | + | + | - | - | - | + | 3 | 0 | 4 | 1 | 38 | Suspend |
| 30 | Child-mother dovetail tenon joint | 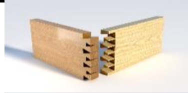 | + | + | + | - | - | - | + | 3 | 0 | 4 | 1 | 38 | Suspend |
| 31 | Centipede joint | 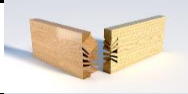 | + | + | + | - | - | - | + | 3 | 0 | 4 | 1 | 38 | Suspend |
| 32 | Heart-shaped joint | 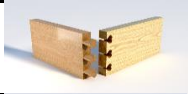 | + | + | + | S | - | - | + | 2 | 1 | 4 | 2 | 32 | Suspend |
| 33 | Keyhole joint | 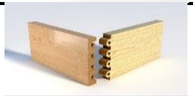 | + | + | + | S | - | - | + | 2 | 1 | 4 | 2 | 32 | Suspend |
| 34 | Oval embedded joint | 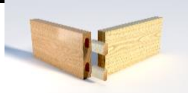 | + | + | S | + | + | + | S | 0 | 2 | 5 | 5 | 1 | Continue |
| 35 | 45 °oval embedded joint | 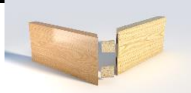 | + | S | S | + | + | + | + | 0 | 2 | 5 | 5 | 1 | Continue |
| 36 | Biscuit embedded tenon joint | 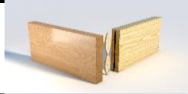 | + | S | S | + | + | + | - | 1 | 2 | 4 | 3 | 24 | Suspend |
| 37 | 45 °biscuit embedded tenon joint | 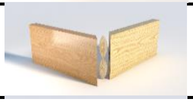 | S | S | S | + | + | + | + | 0 | 3 | 4 | 4 | 8 | Suspend |

| | | | | | | | | | | | | | | | |
|---|---|---|---|---|---|---|---|---|---|---|---|---|---|---|---|
| 38 | Dowels joint | 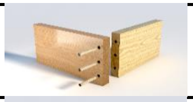 | + | + | S | + | + | + | - | 1 | 1 | 5 | 4 | 8 | Suspend |
| 39 | 45 °dowels joint | 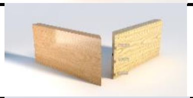 | + | S | - | + | + | + | + | 1 | 1 | 5 | 4 | 8 | Suspend |
| 40 | Half side dowels joint | 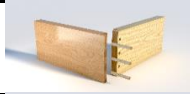 | + | + | - | + | + | + | S | 1 | 1 | 5 | 4 | 8 | Suspend |
| 41 | 45 °Half side dowels joint | 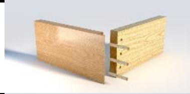 | + | + | S | + | + | + | - | 1 | 1 | 5 | 4 | 8 | Suspend |
| 42 | Square block embedded joint | 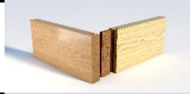 | + | S | S | + | + | + | - | 1 | 2 | 4 | 3 | 24 | Suspend |
| 43 | 45 °Square block embedded joint | 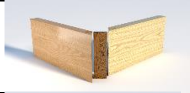 | + | S | S | + | + | + | - | 1 | 2 | 4 | 3 | 24 | Suspend |
| 44 | 45 °dovetail cramp embedded joint | 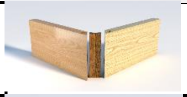 | + | + | S | S | + | S | S | 0 | 4 | 3 | 3 | 24 | Suspend |
| 45 | Dovetail cramp embedded joint | 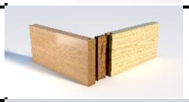 | + | + | S | + | + | S | S | 0 | 3 | 4 | 4 | 8 | Suspend |
| 46 | 45 °half side square block embedded joint | 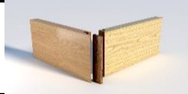 | + | + | S | S | + | S | S | 0 | 4 | 3 | 3 | 24 | Suspend |
| 47 | 45 °cross tenon embedded joint | 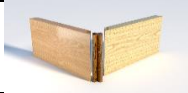 | + | + | S | S | + | S | S | 0 | 4 | 3 | 3 | 24 | Suspend |
| 48 | Triangular block joint | 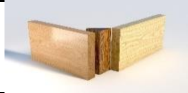 | S | - | - | + | + | + | - | 3 | 1 | 3 | 0 | 53 | Suspend |
| 49 | Hooked block joint | 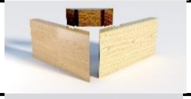 | + | + | S | S | - | - | - | 3 | 2 | 2 | -1 | 58 | Suspend |
| 50 | Half length lap dowels joint | 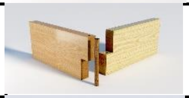 | + | + | S | + | + | + | - | 1 | 1 | 5 | 4 | 8 | Suspend |

| | | | | | | | | | | | | | | | |
|---|---|---|---|---|---|---|---|---|---|---|---|---|---|---|---|
| 51 | Straight mortise dowels joint | 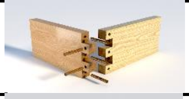 | + | + | + | S | S | + | + | 0 | 2 | 5 | 5 | 1 | Continue |
| 52 | Dovetail tenon dowels joint | 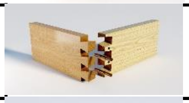 | + | + | + | S | S | S | - | 1 | 3 | 3 | 2 | 32 | Suspend |
| 53 | 45 ° triangular insert joint | 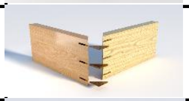 | + | S | + | + | S | + | + | 0 | 2 | 5 | 5 | 1 | Continue |
| 54 | 45 ° dovetail insert joint | 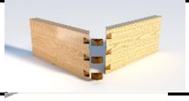 | + | + | + | S | S | S | + | 0 | 3 | 4 | 4 | 8 | Suspend |
| 55 | Fork dovetail tenon joint | 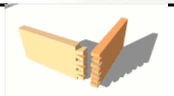 | + | + | + | - | - | S | + | 2 | 1 | 4 | 2 | 32 | Suspend |
| 56 | Pointed dovetail tenon joint | 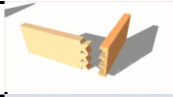 | + | + | + | - | - | S | + | 2 | 1 | 4 | 2 | 32 | Suspend |
| 57 | Pillar block dowels joint | 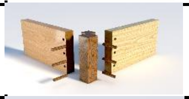 | + | + | + | + | S | - | + | 1 | 1 | 5 | 4 | 8 | Suspend |
| 58 | Double dovetail joint | 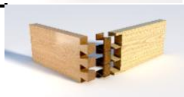 | + | + | + | - | - | - | + | 3 | 0 | 4 | 1 | 38 | Suspend |
| 59 | 45 °pillar block dovetail joint | 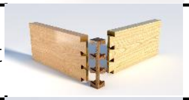 | + | + | S | - | - | - | + | 3 | 1 | 3 | 0 | 53 | Suspend |
| 60 | Inclined hole nail joint | 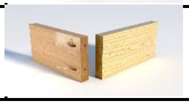 | S | - | - | + | + | + | - | 3 | 1 | 3 | 0 | 53 | Suspend |
