# Supplementary material for: A comprehensive mortise and tenon structure selection method based on Pugh’s controlled convergence and rough Z-number MABAC method
Source: PLoS One. 2023 May 18;18(5):e0283704. doi: 10.1371/journal.pone.0283704 (PMC10194880; doi:10.1371/journal.pone.0283704)
Supplement: S2 Appendix — (PDF) [file pone.0283704.s002.pdf]

Appendix 2: Decisions made by the experts

Decisions made by Expert e1

Alternative	Information\Criteria	C1	C2	C3	C4	C5	C6	C7	C8
A1	Linguistic information	VG	VG	F	G	G	VG	F	G
	Reliability information	VC	VC	VC	VC	VC	VC	VC	VC
A2	Linguistic information	VG	G	G	G	F	EG	VG	EG
	Reliability information	VC	VC	VC	VC	VC	VC	VC	VC
A3	Linguistic information	VG	G	G	G	F	G	EG	F
	Reliability information	VC	VC	VC	VC	VC	VC	VC	VC
A4	Linguistic information	G	G	G	G	G	P	P	F
	Reliability information	VC	VC	VC	VC	VC	C	VC	C
A5	Linguistic information	F	G	F	G	G	P	P	F
	Reliability information	VC	VC	VC	VC	VC	C	C	C
A6	Linguistic information	P	P	F	F	G	G	VG	VG
	Reliability information	VC	VC	VC	VC	VC	VC	VC	VC
A7	Linguistic information	EP	G	G	G	G	VP	F	F
	Reliability information	VC	VC	VC	VC	VC	VC	VC	VC

Decisions made by Expert e2

Alternative	Information\Criteria	C1	C2	C3	C4	C5	C6	C7	C8
A1	Linguistic information	G	P	P	F	P	G	G	VG
	Reliability information	VC	VC	VC	VC	VC	VC	VC	VC
A2	Linguistic information	VG	P	P	F	P	EG	VG	EG
	Reliability information	VC	VC	VC	VC	VC	VC	VC	VC
A3	Linguistic information	F	F	P	F	P	F	G	G
	Reliability information	VC	VC	VC	VC	VC	VC	VC	VC
A4	Linguistic information	F	G	G	G	G	P	F	EP
	Reliability information	VC	VC	VC	VC	VC	VC	VC	VC
A5	Linguistic information	F	G	G	G	G	P	F	EP
	Reliability information	VC	VC	VC	VC	VC	VC	VC	VC
A6	Linguistic information	F	F	F	F	F	P	F	EP
	Reliability information	VC	VC	VC	VC	VC	VC	VC	VC
A7	Linguistic information	F	G	G	F	G	P	F	EP
	Reliability information	VC	VC	VC	VC	VC	VC	VC	VC

Decisions made by Expert e3

Alternative	Information\Criteria	C1	C2	C3	C4	C5	C6	C7	C8
A1	Linguistic information	VG	VG	VG	VG	VG	VG	F	VG
	Reliability information	VC	VC	VC	VC	VC	VC	VC	VC
A2	Linguistic information	EG	VG	VG	VG	VG	VG	VG	VG
	Reliability information	VC	VC	VC	VC	VC	VC	VC	VC
A3	Linguistic information	F	F	F	F	G	F	G	F
	Reliability information	VC	VC	VC	VC	VC	VC	VC	VC
A4	Linguistic information	P	F	F	F	P	P	P	P
	Reliability information	VC	VC	VC	VC	VC	VC	VC	VC
A5	Linguistic information	P	P	F	F	F	P	G	F
	Reliability information	VC	VC	VC	VC	VC	VC	VC	VC
A6	Linguistic information	G	F	F	F	F	G	G	G
	Reliability information	VC	VC	VC	VC	VC	VC	VC	VC
A7	Linguistic information	P	P	F	F	G	P	G	P
	Reliability information	VC	VC	VC	VC	VC	VC	VC	VC

Decisions made by Expert e4

Alternative	Information\Criteria	C1	C2	C3	C4	C5	C6	C7	C8
A1	Linguistic information	VG	VG	EG	G	EG	F	F	F
	Reliability information	VC	VC	VC	VC	VC	VC	VC	VC
A2	Linguistic information	VG	G	F	G	F	EG	VG	VG
	Reliability information	VC	VC	VC	VC	VC	VC	VC	VC
A3	Linguistic information	VG	P	F	F	P	VG	EG	VG
	Reliability information	VC	VC	VC	VC	VC	VC	VC	VC
A4	Linguistic information	P	EG	EG	VG	VG	G	P	F
	Reliability information	VC	VC	VC	VC	VC	VC	VC	VC
A5	Linguistic information	VG	F	VG	VG	VG	F	EG	G
	Reliability information	VC	VC	VC	VC	VC	VC	VC	VC
A6	Linguistic information	F	G	VG	VG	VG	VG	G	VG
	Reliability information	VC	VC	VC	VC	VC	VC	VC	VC
A7	Linguistic information	G	VG	VG	VG	VG	G	VG	G
	Reliability information	VC	VC	VC	VC	VC	VC	VC	VC

Decisions made by Expert e5

Alternative	Information\Criteria	C1	C2	C3	C4	C5	C6	C7	C8
A1	Linguistic information	G	G	F	G	F	G	G	G
	Reliability information	C	C	C	C	C	C	C	C
A2	Linguistic information	P	P	P	P	P	G	G	G
	Reliability information	C	C	C	C	C	C	C	C
A3	Linguistic information	G	G	G	G	G	VG	VG	VG
	Reliability information	C	C	C	C	C	C	C	C
A4	Linguistic information	G	G	G	G	G	F	F	P
	Reliability information	C	C	C	C	C	C	C	C
A5	Linguistic information	G	F	F	G	F	F	F	F
	Reliability information	C	C	C	C	C	C	C	C
A6	Linguistic information	F	G	F	G	F	G	G	G
	Reliability information	C	C	C	C	C	C	C	C
A7	Linguistic information	G	G	G	G	G	F	F	F
	Reliability information	C	C	C	C	C	C	C	C

Decisions made by Expert e6

Alternative	Information\Criteria	C1	C2	C3	C4	C5	C6	C7	C8
A1	Linguistic information	G	F	F	G	G	VG	F	G
	Reliability information	C	M	M	C	C	C	C	C
A2	Linguistic information	G	G	G	G	G	VG	F	G
	Reliability information	C	M	M	C	C	C	C	C
A3	Linguistic information	G	VG	VG	G	F	G	G	G
	Reliability information	C	C	C	C	C	C	C	C
A4	Linguistic information	G	VG	VG	G	F	G	G	G
	Reliability information	C	C	C	C	C	C	C	C
A5	Linguistic information	G	VG	VG	G	F	G	G	G
	Reliability information	C	C	C	C	C	C	C	C
A6	Linguistic information	F	G	G	F	G	G	F	G
	Reliability information	M	C	C	C	C	C	C	C
A7	Linguistic information	F	G	G	F	G	G	F	G
	Reliability information	M	C	C	C	C	C	C	C

Decisions made by Expert e7

Alternative	Information\Criteria	C1	C2	C3	C4	C5	C6	C7	C8
A1	Linguistic information	G	EG	EG	EG	EG	EG	VG	EG
	Reliability information	AC	AC	AC	AC	AC	AC	AC	M
A2	Linguistic information	G	VG	G	EG	G	VG	F	VG
	Reliability information	AC	AC	C	AC	VC	VC	AC	M
A3	Linguistic information	G	VG	G	EG	G	VG	F	VG
	Reliability information	AC	AC	C	AC	VC	VC	AC	M
A4	Linguistic information	G	G	G	F	G	F	F	F
	Reliability information	AC	AC	C	C	VC	C	AC	M
A5	Linguistic information	G	F	F	F	G	F	G	F
	Reliability information	AC	AC	C	VC	C	C	AC	M
A6	Linguistic information	G	P	P	P	P	EG	VG	EG
	Reliability information	AC	AC	AC	AC	AC	AC	AC	M
A7	Linguistic information	G	VG	VG	G	G	P	EG	F
	Reliability information	AC	AC	VC	C	C	C	AC	M

Decisions made by Expert e8

Alternative	Information\Criteria	C1	C2	C3	C4	C5	C6	C7	C8
A1	Linguistic information	P	P	P	P	P	G	G	G
	Reliability information	C	C	C	C	C	C	C	C
A2	Linguistic information	P	P	P	P	P	G	G	G
	Reliability information	C	C	C	C	C	C	C	C
A3	Linguistic information	P	P	P	P	P	G	G	G
	Reliability information	C	C	C	C	C	C	C	C
A4	Linguistic information	G	G	G	G	G	F	F	F
	Reliability information	C	C	C	C	C	C	C	C
A5	Linguistic information	G	G	G	G	G	F	F	F
	Reliability information	C	C	C	C	C	C	C	C
A6	Linguistic information	P	P	G	G	G	G	F	G
	Reliability information	C	C	C	C	C	C	C	C
A7	Linguistic information	G	G	G	G	G	F	F	F
	Reliability information	C	C	C	C	C	M	M	M

Decisions made by Expert e9

Alternative	Information\Criteria	C1	C2	C3	C4	C5	C6	C7	C8
A1	Linguistic information	EG	EG	EG	EG	EG	VG	F	G
	Reliability information	U	VC	AC	C	C	C	AC	U
A2	Linguistic information	EG	G	F	EG	G	EG	G	EG
	Reliability information	U	C	M	C	C	VC	AC	AC
A3	Linguistic information	EG	G	F	EG	G	EG	G	EG
	Reliability information	U	C	M	C	C	VC	AC	AC
A4	Linguistic information	EG	EG	P	F	F	EG	VG	F
	Reliability information	U	C	U	M	U	C	AC	U
A5	Linguistic information	EG	EG	P	F	F	EG	EG	F
	Reliability information	U	C	U	M	U	C	AC	U
A6	Linguistic information	EG	P	P	P	F	EG	VG	EG
	Reliability information	U	C	U	M	U	C	VC	VC
A7	Linguistic information	EG	P	P	G	F	G	EG	EG
	Reliability information	U	C	U	M	U	C	AC	M
